# Mitomycin-Treated Endothelial and Smooth Muscle Cells Suitable for Safe Tissue Engineering Approaches

**DOI:** 10.3389/fbioe.2022.772981

**Published:** 2022-03-11

**Authors:** Irina Zakharova, Shoraan Saaya, Alexander Shevchenko, Alena Stupnikova, Maria Zhiven', Pavel Laktionov, Alena Stepanova, Alexander Romashchenko, Lyudmila Yanshole, Alexander Chernonosov, Alexander Volkov, Elena Kizilova, Evgenii Zavjalov, Alexander Chernyavsky, Alexander Romanov, Andrey Karpenko, Suren Zakian

**Affiliations:** ^1^ The Federal Research Center Institute of Cytology and Genetics, The Siberian Branch of the Russian Academy of Sciences, Novosibirsk, Russia; ^2^ E.N. Meshalkin National Medical Research Center, Ministry of Health of the Russian Federation, Novosibirsk, Russia; ^3^ Institute of Chemical Biology and Fundamental Medicine, The Siberian Branch of the Russian Academy of Sciences, Novosibirsk, Russia; ^4^ Deparment of Natural Science, Novosibirsk State University, Novosibirsk, Russia; ^5^ International Tomography Center,The Siberian Branch of the Russian Academy of Sciences, Novosibirsk, Russia

**Keywords:** endothelial cells, smooth muscle cells, mitomycin C, vascular patch, tissue-engineered vascular graft, polycaprolactone

## Abstract

In our previous study, we showed that discarded cardiac tissue from the right atrial appendage and right ventricular myocardium is an available source of functional endothelial and smooth muscle cells for regenerative medicine and tissue engineering. In the study, we aimed to find out what benefits are given by vascular cells from cardiac explants used for seeding on vascular patches engrafted to repair vascular defects *in vivo*. Additionally, to make the application of these cells safer in regenerative medicine we tested an *in vitro* approach that arrested mitotic division to avoid the potential tumorigenic effect of dividing cells. A tissue-engineered construction in the form of a patch based on a polycaprolactone-gelatin scaffold and seeded with endothelial and smooth muscle cells was implanted into the abdominal aorta of immunodeficient SCID mice. Aortic patency was assessed using ultrasound, MRI, immunohistochemical and histological staining. Endothelial and smooth muscle cells were treated with mitomycin C at a therapeutic concentration of 10 μg/ml for 2 h with subsequent analysis of cell proliferation and function. The absence of the tumorigenic effect of mitomycin C-treated cells, as well as their angiogenic potential, was examined by injecting them into immunodeficient mice. Cell-containing patches engrafted in the abdominal aorta of immunodeficient mice form the vessel wall loaded with the appropriate cells and extracellular matrix, and do not interfere with normal patency. Endothelial and smooth muscle cells treated with mitomycin C show no tumorigenic effect in the SCID immunodeficient mouse model. During *in vitro* experiments, we have shown that treatment with mitomycin C does not lead to a decrease in cell viability. Despite the absence of proliferation, mitomycin C-treated vascular cells retain specific cell markers, produce specific extracellular matrix, and demonstrate the ability to stimulate angiogenesis *in vivo*. We pioneered an approach to arresting cell division with mitomycin C in endothelial and smooth muscle cells from cardiac explant, which prevents the risk of malignancy from dividing cells in vascular surgery. We believe that this approach to the fabrication of tissue-engineered constructs based on mitotically inactivated cells from waste postoperative material may be valuable to bring closer the development of safe cell products for regenerative medicine.

## Introduction

It is estimated that 6-8 out of 1,000 children are born with congenital vascular defects (Sun et al., 2015; Liu et al., 2019). After the first vascular reconstructive surgery, many patients have a high probability of reoperation during their lifetime due to conduit dysfunction (stenosis, thrombosis, and aneurysm) or its inability to grow in a growing body. A promising approach in regenerative medicine is designing tissue-engineered substitutes for vessels seeded with autologous endothelial and smooth muscle cells that are the main part of the vessel wall (Pashneh-Tala et al., 2016; Skovrind et al., 2019). It is expected that tissue-engineered vascular substitutes containing cells will be as close as possible to physiological ones in their properties and will reduce long-term negative consequences in the postoperative period.

Even 30 years after the first tissue-engineered vascular graft was developed, the design of new vascular substitutes, including small-diameter ones, is still relevant (Weinberg and Bell, 1986; Skovrind et al., 2019). A recent meta-study showed that tissue-engineered vascular grafts (TEVG) that had undergone long-term recellularization showed the best patency rates (Skovrind et al., 2019). Cell colonization has been shown to reduce the thrombogenicity of small-diameter tissue-engineered vessels (SD-TEVG) and improve long-term patency outcomes (Sánchez et al., 2018; Fang et al., 2021).

The cell sources for vascular graft colonization are of fundamental importance for the subsequent correct construct integration into the surrounding recipient tissues. A number of studies currently offer various sources of clinically applicable postnatal endothelial (Winiarski et al., 2011; DeLeve, 2013; Jia et al., 2013; Zhou et al., 2014) and smooth muscle (Churchman and Siow, 2009; Lu et al., 2013) cells. As vascular cells are heterogeneous in their molecular characteristics depending on the vessel types, tissues, and organs (Aird, 2012; Nolan et al., 2013), for successful regeneration, it is important to use appropriate vascular cells, which correspond to a specific molecular genetic pattern. Transcriptomic analysis revealed specific pathways, transporters, and cell-surface markers expressed in the endothelium of each organ (Jambusaria et al., 2020). Recently identified 37 differentially expressed immune-related genes across ECs from different anatomic origins (Gunawardana et al., 2021). Endothelial heterogeneity contributes to the response of an organism to organ and tissue transplantation. In this regard, the available sources of autologous vascular cells in a therapeutically sufficient amount are still an open question.

In our previous study, we found that surgical discarded cardiac tissue as a right atrial appendage and right ventricular myocardium is an available source for functional endothelial and smooth muscle cells potentially suitable for vascular tissue engineering (Zakharova et al., 2017). We derived endothelial and smooth muscle cell cultures from human cardiac post-operative explants. Explants were enzymatically treated and released heterogeneous primary cells were grown in defined endothelial and smooth muscle cultural media on collagen IV-coated surfaces. The population of endothelial cells was further enriched by immunomagnetic sorting for CD31. The cells demonstrated their functional properties *in vitro* as part of tissue-engineered constructs made from polycaprolactone (PCL) and chitosan. In our current work, vascular cells from cardiac explants were tested *in vivo* on a model of immunodeficient mice as part of an aortic patch made from polycaprolactone-gelatin mix by electrospinning. We also paid attention to the fact that dividing cells used in tissue engineering may cause malignancy (Forsyth et al., 2004; Rea et al., 2006; Jiang, 2010; Schmedt et al., 2012; Meier et al., 2013; Ravi and Patel, 2013; Wagner et al., 2017; Sato et al., 2019; Heydari et al., 2020; Wuputra et al., 2020; Li et al., 2021). We proposed a method for mitotic inactivation of the dividing cells to avoid their potential tumorigenic effect. We showed that mitotically inactivated vascular cells from cardiac explants retain their functional properties and viability even after cell division arrest.

This work provides the basis for a conceptually new approach to regenerative tissue engineering methods using endothelial (EC) and smooth muscle (SMC) cells. Described previously EC and SMC from cardiac explants is promising autologous patient-specific cells, especially for reoperation. Being isolated from postoperative material the cells are easily expanded and cryopreserved until the patient needs to eliminate the cardio-vascular defect with the tissue-engineered graft containing cells. These cells can also be considered for use as allogeneic, for example, if the donor and recipient HLA genotypes match. Mitotic inactivation before or after cell seeding on a synthetic scaffold eliminates the potential risk of malignancy from transplanted cells. We suggest that the application of cell-seeded patient-specific tissue-engineered grafts may reduce the need for subsequent surgical intervention to replace a vascular prosthesis due to their dysfunction or inability to grow.

## Materials and Methods

### Ethical Statements

All procedures with human material were approved by the ethical committee of Meshalkin National Medical Research Centre (permit No. 45 by 26.12.2014), and all donors signed an informed consent form. The animal tests conducted in an SPF vivarium were approved by the Institute of Cytology and Genetics ethical board (permit No. 22.4 by 30.05.2014).

### Scaffold Fabrication

Synthetic biodegradable scaffolds from polycaprolactone (PCL) (Sigma, #44074-5G) with gelatin (Sigma, #G2500-100G) and a low-permeable inner layer were prepared by electrospinning according to a previously published work (Popova et al., 2015). Synthetic scaffolds with a low-permeable layer were made by sequentially applying 3 layers of a matrix from polymer solutions of a certain composition using the electrospinning method, as described in the patent RU2572333. The thickness of the scaffold was ∼80 µm. The first inner matrix layer and the next low-permeable layer have about 10 µm each. Low-permeable layer was produced from 1.5% w/v PCL with 3.5% w/v gelatin in hexafluoroisopropanol, while remaining scaffold from 4.5% w/v PCL and 0.5% w/v gelatin in the same solvent. The electrospinning conditions include the solution feed rate of 1.2 ml/h, the voltage of 20–25 kV, the cylindrical collector rotation speed of 300 rpm, and the distance between the capillary and the collector of 20 cm. After fabrication, the scaffolds were removed from the collector and dried in a vacuum. After preparation, the matrix was incubated in phosphate buffer for at least an hour and then cross-linked with glutaraldehyde, as described previously (Chernonosova et al., 2018).

The scaffold microstructure was examined by scanning electron microscopy (SEM) as described earlier (Chernonosova et al., 2017). The fiber diameter was assessed from the SEM images according to ISO 7198:1998.

**FIGURE 1:**
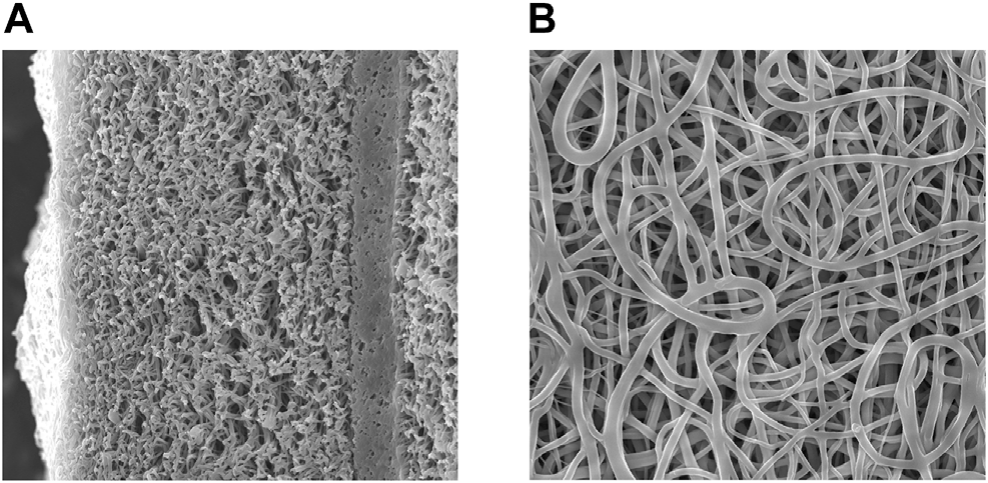
Scanning electron microscopy image of a scaffold slice with a low-permeability layer **(A)** magnification 1,000X, the inner surface of the scaffold **(B)** magnification 2.500X.

The morphology of the resulting scaffolds was studied using scanning electron microscopy. The image was obtained on a JSM-6460 LV scanning electron microscope (Jeol, Japan). This part of the work was carried out at the Department of Physicochemical Methods of the Institute of Catalysis, SB RAS. Electron microscopy images of the scaffold are shown in Figure 1.

According to SEM images, after exposure to phosphate buffer for an hour, the scaffolds have a fiber diameter of 0.5 ± 0.1 μm and a denser layer with low permeability. It was shown the low-permeable inner layer does not affect the strength of the tubes, displaying a tensile strength of 2.2÷2.5 MPa, and a thread breaking strength (suture retention strength) of 160 ± 20 g of force. A low-permeable inner layer reduces the material permeability for water from 5 to 10 ml to 0.1 ÷ 0.2 ml.

### Cell Cultivation and Seeding on Polycaprolactone Patch

Endothelial and smooth muscle cells were isolated and cultivated as described in our previous work (Zakharova et al., 2017). For experiments with MMC, cells growing on plastics were detached with TrypLE, washed with PBS, centrifuged at 300 g for 5 min followed by dissolving the pellet in a growth medium containing 10 μg/ml MMC (Sigma, #M4287) (1 ml per 5 × 10^5^ cells) and incubated for 2 h at 37°C in 5% CO_2_, vortexing occasionally. The cells were then washed 3 times with PBS (Biolot, #2.1.1) and seeded in a plastic tissue culture flask with a growth medium.

To prepare a tissue-engineered construct a PCL patch of 2 cm^2^ was seeded with 3 × 10^5^ per cm^2^ of cells. First, on one side of the patch, we plated smooth muscle cells in SmGM-2 medium (Lonza, #CC-3182). As a control, PCL patches without cells were placed into the same medium. After a day, the patch was turned over, and the other side was seeded with endothelial cells in a mixture of EGM-2 (Lonza, #CC-3162) + SmGM-2 media. Control PCL patches with no cells were placed in the same medium. The patches seeded with cells and unseeded control patches were cultured for another 5 days. The cells on the patches and unseeded control patches were incubated at 37°C in 5% CO_2_.

We plated the cells on a scaffold for 6 days to allow them to form an extracellular matrix (ECM), which creates the physiological cellular microenvironment necessary for proper integration of the patch into the surrounding tissues and adequate colonization of the recipient cells. The ECM contains internal biochemical and mechanical signals that contribute to the formation of the correct hierarchy of recipient cells, populating the patch after engraftment (Hussey et al., 2018; Niklason, 2018; Zhu et al., 2019).

### Graft Implantation

The animals were kept in single-sex family groups of 2–5 mice in individually ventilated cages using the Optimice system (Animal Care Systems, Centennial, CO, United States). The mice were maintained under controlled conditions: temperature, 22–26°C; relative humidity, 30–45%; and 12/12 light/dark periods with dawn at 02:00. Standard V1534-300 ssniff^®^ diet (ssniff Spezialdiäten GmbH, Soest, Germany) and reverse osmosis water, enriched with a mineral mixture Severyanka (St-Peterburg), were provided to the animals ad libitum.

We implanted 3D patches with and without cells in the abdominal aorta of 35 female SCID mice, 6–7 weeks old, average body weight 24 ± 4 g. The mice were divided into 2 groups. The experimental group included 23 mice implanted with patches pre-populated with cells. The control group consisted of 12 mice with implanted patches without cells. SCID mice were anesthetized with isoflurane and subsequently opened under standard sterile conditions. The abdominal aorta below the renal arteries up to the aortic bifurcation was isolated by a midline laparotomy approach. The aorta below the renal arteries and above the bifurcation was taken on holders (8/0 Prolene sutures). Next, the aorta was clamped with holders, and a longitudinal aortotomy of the abdominal aorta up to 0.5 mm in length was performed. Mitomycin C was not used during the aortotomy or patch implantation. Then a 0.5 × 0.5 mm patch was sewn into the aortotomy defect with 4 interrupted sutures using Premilene 10/0 suture. The patch implantation stages are shown in Supplementary Figure S1A. After confirmation of blood flow and hemostasis following removal of the clamp, the wound area was closed by layer-by-layer suturing of the anterior abdominal wall and skin with 6\0 threads. Prior, during, and after surgery, mice did not receive anticoagulants or antiplatelet agents.

Wall thickness of vascular patches before and 24 weeks after implantation was measured by electronic outside micrometer (0–25 mm, 0.001), Schut Geometrical Metrology, 908.750.

### *In vivo* Microcomputed Tomography and Ultrasonography

Abdominal aortic patency was assessed at 2, 4, 12, and 24 weeks after surgery. The study was carried out using magnetic resonance imaging (MRI) on an ultra-high-field tomograph BioSpec 117/16 USR (Bruker, Germany)—11.7 Tesla. F T1-weighted images were obtained by the FLASH method (Fast Low Angele SHot) with pulse sequence parameters (TE = 2.5 ms, TR = 200 ms), and an image (size 3 × 3 cm; on a matrix of 128 × 128 points; slice thickness 0.31 mm and distance between slices 0.33 mm).

Ultrasound examination was carried out at the same time. Under anesthesia with tiletamine (Zoletil 50, 5 mg/kg) and xylazine (XylaVet, 3 mg/kg) we assessed the permeability of the aorta, blood flow velocity in the area of the implanted patch after 2, 4, 12, and 24 weeks using a Vivid 4 apparatus with a linear vascular sensor (GE Medical Systems Israel Ltd., Ultrasound).

### Immunofluorescent Staining of Cells in Culture Plastic and Tissue-Engineered Constructs

MMC-treated ECs and SMCs on plastic dishes were fixed with 4% FA (formaldehyde) (Sigma, #F8775-500 ML) for 10 min, permeabilized with 0.05% Triton X-100 (Sigma, #T8787-250 ML) for 10 min, and blocked with 1% BSA (bovine serum albumin) (VWR Life Science AMRESCO, #0332-100G).

Patch material at the 2nd, 4th, 12th, 24th weeks was collected by the mouse aorta transverse excision proximal and distal to the patch. After longitudinal dissection of the aorta posterior wall, the collected material was washed twice in PBS, fixed in 4% FA for 1 h, permeabilized with 1% Triton X-100 for 30 min, and blocked with 1% BSA.

The cells were stained with primary antibodies overnight at 4°C, washed with PBS, and incubated with secondary antibodies for 1 h at room temperature. The stained cells were analyzed with an inverted fluorescence microscope (Nikon Ti-E) using Nikon AR software. The cells in the tissue-engineered construct were analyzed using an LSM 780 confocal microscope (Zeiss) at the Common Facilities of Microscopic Analysis of Biological Objects, ICG SB RAS.

The following primary antibodies were used:

anti-human CD31 (DAKO, #M0823, 1:50), mouse IgG1; anti-mouse CD31 (Biolegend, #102502), rat IgG2a; anti-α-SMA (DAKO, #M0851, 1:50), mouse IgG2a; anti-smooth muscle myosin heavy chain 11 (Abcam, #ab82541, 1:500), rabbit polyclonal; anti-Von Willebrand factor (Abcam, #ab6994, 1:200), rabbit polyclonal; anti-fibronectin (Abcam, #ab6328, 1:200), mouse IgG1; anti-elastin (Abcam, #ab21610, 1:200), rabbit polyclonal; anti-collagen IV (Life Span, #LS-C79603, 1:200) mouse IgG1.

The following secondary antibodies were used:

Alexa Fluor 488 goat anti-mouse IgG1 (Life Technologies, #A21121, 1:400), Alexa Fluor 488 goat anti-rabbit IgG H + L (Life Technologies, #A11008, 1:400), Alexa Fluor 488 goat anti-rat IgG H + L (Life Technologies, #A11006, 1:400), Alexa Fluor 568 goat anti-mouse IgG1 (Life Technologies, #A21124, 1:400), Alexa Fluor 568 goat anti-mouse IgG2a (Life Technologies, #A21134, 1:400), Alexa Fluor 568 goat anti-rabbit IgG H + L (Life Technologies, #A11011, 1:400).

Representative images demonstrating negative control for autofluorescence with no primary antibody are shown in Supplementary Figure S2.

### Preparation of Cryosections. Hematoxylin and Eosin Staining

The patch material, explanted and fixed as for Immunofluorescent staining, was washed from the fixative at room temperature on a shaker in 125 mM glycine (Helicon, #Am-O167-0.5)/x1 PBS solution for 30 min, and then two times in PBS for 30 min. To restore the tissue structure, samples were incubated for 1 h in 20% sucrose at room temperature. They were then incubated overnight in 30% sucrose at + 4°C. The samples were embedded in O.C.T.-compound (Sakura, Tissue Tek, #4583), cooled to -22°C, 10 μm cryosections were made using a Cryostat HM 550 instrument on Superfrost Plus, Menzel-Glasses (Thermo Scientific, #J1800AMNZ). Histological preparations were stained with hematoxylin and eosin. Stained sections were examined by light microscopy using an AXIO Lab. A1 ZEISS microscope. The obtained microscopic images were used to assess the structure of the endothelial layer, the presence of cells in construct, patch fiber integrity, and the composition of the outer fibrous capsule.

### Mitomycin C Assay by HPLC–MS/MS

A set of Mitomycin C (MMC) calibration solutions at various concentrations (1, 2, 5, 10, 50, 100, 250, and 500 ng/ml) was prepared from a standard stock solution of 2 mg/ml MMC by dilution with deionized water. The calibration solutions were analyzed via HPLC-MS/MS (MRM) to generate a standard curve. Various metabolites including MMC were extracted in H_2_O: methanol (1: 4) from 5 EC and 5 SMC samples containing 1.3 to 2.5 million cells each. At first, 300 µl of H_2_O was added to each tube with the cell sample followed by two cycles of freezing in liquid nitrogen and thawing in cold water. All tubes were vortexed between freeze-thaw cycles. Then 1.2 ml of cold (−20°C) methanol was added to each tube. The mixture was shaken vigorously for 10 min and left at −20°C for 30 min. Then the mixture was centrifuged at 16,100 g, + 4°C for 30 min to obtain a protein pellet and a methanol-H_2_O layer. This upper aqueous layer was collected and lyophilized using an RVC 2-25 CD rotary vacuum concentrator (Martin Christ, Germany). The resulting protein-free extracts were used for LC-MS measurements.

The extracts for LC-MS analysis were re-dissolved in 100 µl of deionized water. The LC separation was performed on an Agilent 1,200 Series chromatograph (Agilent Technologies, United States) using Acclaim Polar Advantage II column (3 × 150 mm, 3 µm) with an Acclaim guard cartridge (Dionex, Germany). Solvent A consisted of 0.1% formic acid solution in H_2_O, solvent B consisted of 0.1% formic acid solution in acetonitrile. The solvent B gradient was 10–55% (0–12 min), 55–95% (12–13 min), 95% (13–16 min), 95–10% (16–16.1 min), 10% (16.1–24 min); at the flow rate of 0.4 ml/min and the sample injection volume of 25 µl. The mass spectra were recorded at Agilent 6,410 Triple Quadrupole (Agilent Technologies, United States) in a positive mode. The instrument parameters were: electrospray voltage 3,500 V; nebulizer pressure 35 psi; dry gas flow 8 L/min; dry gas temperature 325°C, fragmentor voltage 100 V; collision gas nitrogen; collision energy 15 V, dwell time 500 ms, Delta EMV 400 V, mass transition m/z 335 to 242 for mitomycin C.

### XTT Proliferation Assay

The proliferative activity of endothelial and smooth muscle cells non-treated and treated with MMC was assessed as previously described (Zakharova et al., 2017) by an XTT test with 2,3-bis- (2-methoxy-4-nitro-5- sulfophenyl) -2H-tetrazolium-5-carboxanilide (Roche, 11465015001, United States) according to the manufacturer’s instructions at 22 h after the reagents were added. The experiment was performed with exactly the same number of cells, which is 10^4^ per well in a 96-well plate. Before planting, cells were counted using Countess 2 Cell Counter (Thermo). For each point, the experiments were carried out in three replicates, with three wells of cells in each replication. Thus, nine values were analyzed for each point. XTT test values were recorded once a day within 8 days after cells were plated.

### Cell Viability Assay

The viability of cells was assessed by staining with the Propidium Iodide/Annexin V*FITC kit (BioLegend, #640914) according to the manufacturer’s protocol and subsequent flow cytometry. The day before the analysis, cells were treated with MMC. Untreated cells were used as a control. The percentage of living, apoptotic, and necrotic cells was determined by flow cytometry for 10^4^ events using a FACS Canto II device (software—FACS Diva 7.0).

The viability of MMS-treated and untreated cells seeded on a PCL patch was assessed by staining with TMRM (tetramethylrhodamine, Thermo, #T668) and Annexin V*FITC (BioLegend, #640914) followed by life-imaging. TMRM is sequestered by active mitochondria and stains only living cells, while Annexin V*FITC identifies apoptotic cells. For the convenience of subsequent counting, cells were seeded on a patch at a low concentration (10^4^ cells per 1 cm^2^ of the patch). In 5 days, cells were stained with TMRM and Annexin V*FITC according to the manufacturer’s protocols with modifications. First, the nuclei were stained with a NucBlue™ Live ReadyProbes™ Reagent (Thermo, #R37605) for 1 h under normal cell growth conditions in a CO_2_ incubator.

After that, the patch was washed 1 time with PBS and placed in a 48-well with 500 μl Annexin V Binding Buffer (BioLegend, #640914), 1 μl of 50 μM TMRM, and 25 μl of Annexin V*FITC for 30 min at room temperature in the dark. Stained cells were visualized in the Annexin V Binding Buffer, using an LSM 780 NLO confocal microscope (Zeiss) at the Common Facilities of Microscopic Analysis of Biological Objects, ICG SB RAS. For each sample, the percentage of TMRM- and Annexin V-positive cells containing nuclei in 10 fields of view was calculated.

### Karyotyping of Cells

G-banding karyotyping was performed at passage 8 of EC and SMC as previously described (Malakhova et al., 2020). We analyzed 50 metaphase spreads using Nikon microscope TiE with Lucia Cytogenetics software.

To study the karyotype stability of MMC-treated cells, molecular karyotyping was carried out which is a study of copy number variations in genomic DNA by sequencing. Molecular karyotyping was performed by Genoanalytica (https://www.genoanalytica.ru). The analysis allows identifying microinsertions and microdeletions of 50,000 base pairs or longer. The karyotype was determined in 4 groups of samples, namely 1) endothelial cells treated with MMС (n = 3), 2) untreated endothelial cells (n = 3), 3) smooth muscle cells treated with MMС (n = 3), 4) untreated smooth muscle cells (n = 2). Genomic DNA was isolated by cell lysis followed by purification on glass fiber filters (QIAamp DNA Mini Kit reagents, Qiagen), then fragmented to 200 bp and used to prepare genomic libraries with NEB II Ultra reagents (New England Biolabs) for subsequent PCR amplification during 8 cycles. The nucleotide sequences were determined at the HiSeq 1,500 instrument (Illumina) with HiSeq Rapid SBS Kit v2 reagents (Illumina). The resulting 1,866,1932 reads are mapped to the reference human genome GRCh37/hg19 version. Rearrangements were identified as genome regions 5,000 kb or longer with abnormal read coverage determined by biased up or down coverage regression function at 1.5 times or more. Molecular karyotyping raw data is available at https://doi.org/10.6084/m9.figshare.17702513.

Statistical analysis of the number of detected CNVs was carried out taking into account the statistical weights of rearrangements in each group.

### Angiogenic Potential Evaluation for Mitotically Inactivated Endothelial and Smooth Muscle Cells *in vivo* in a Matrigel Plug Model

An *in vivo* Matrigel angiogenesis assay was conducted as previously described (Zakharova et al., 2017). Female immunodeficient (severe combined immunodeficiency, SCID) mice (6–8 weeks, 22–28 g) were used for experiments. Animals were kept in the SPF-vivarium of the ICG SB RAS (Novosibirsk, Russia). In the experiment, 17 mice were divided into groups as follows:

group 1. MMC treated ECs + SMCs, 5 mice; group 2. Untreated MMC ECs + SMCs, 5 mice; group 3. Untreated MMC ECs + SMCs stained with the intravital mitochondrial dye MitoTracker Deep Red FM (Invitrogen, M22426), 2 mice; group 4. Control—matrigel without cells, 5 mice.

Endothelial and smooth muscle cells (5 × 10^5^ each) in 50 μl of growth medium were mixed with 50 μl of Matrigel (Corning, #354234) immediately before injection. The total mixture in a volume of 100 μl was injected to mice subcutaneously into the abdominal region for 14 days. Isolectin B4 conjugated with biotin (Invitrogen, I21414) was injected into the Matrigel implant area 10 min before mouse sacrifice.

A Kodak *In-Vivo* Multispectral Imaging System was used to visualize cells stained with an *in vivo* mitochondrial dye (MitoTracker Deep Red FM).

Matrigel implants were removed, fixed in 4% PFA, frozen in Tissue-Tek O.C.T. Compound. Then, 10 μm cryosections were obtained from a vascularized implant on a Leica HM550 cryotome (Leica, Germany). The sections were incubated with a streptavidin-Cy3 fluorochrome conjugate (Sigma-Aldrich, S6402-1ML). Isolectin-positive vessels were imaged by a Nikon Ti-E microscope in 10 fields of view (FOV). Comparison between groups was carried out according to three parameters: the total vessels length, the average vessel length, and the total number of junctions in vasculature.

Imaged vasculature was assessed by the Angio Tool software (Zudaire et al., 2011), the results were compared by the Wilcoxon test with Bonferroni’s correction in R software version 3.5.1 (R Core Team, 2021). Representative images were taken using an LSM 780 NLO confocal microscope (Zeiss) at the Common Facilities of Microscopic Analysis of Biological Objects, ICG SB RAS.

### Tumorigenicity Assay of Mitotically Inactivated Endothelial and Smooth Muscle Cells *in vivo*

The experiment was carried out at the Common Facilities Centre “SPF-vivarium of the ICG SB RAS” using female homozygous immunodeficient SCID mice at the age of 6–8 weeks. HEK293FT cells were used as a positive tumorigenic control (Stepanenko and Dmitrenko, 2015). Experimental mice groups are described above in Results.

We mixed 100 μl of Matrigel (BD Biosciences, United States) with 1 × 10^6^ cells in 100 μl of growth medium immediately before injection. We injected 200 μl of this mixture into mice subcutaneously into the abdominal region and left for 3 months. The positive control group was sacrificed earlier than the indicated period because tumor formation tends to necrotic changes.

Tissue samples taken at the site of injection were fixed in neutral (buffered, pH 7.6-7.8) 10% formalin and embedded in paraffin using standard techniques (Vaskova et al., 2015). Skin samples were oriented orthogonally to the epidermis. Series of 7–10 µm sections were mounted on Superfrost Plus Menzel-Glasses (Thermo Scientific, #J1800AMNZ) and stained as previously described (Vaskova et al., 2015).

Paraffin-embedded tissue sections (7–10 mm) were prepared and stained according to the previously described protocols (Vaskova et al., 2015).

Sample preparation, image capturing, and processing was performed at the Common Facilities Center of Microscopic Analysis of Biological Objects, ICG SB RAS (https://ckp.icgen.ru/ckpmabo/).

### Statistics

Statistical significance in value differences among experimental groups, we calculated by univariate analysis of variance (ANOVA) in Microsoft Excel (Microsoft Office 2013) software and Wilcoxon’s T-test in R version 3.5.1 (R Core Team, 2021). Bonferroni correction was applied for multiple comparisons. In the case of a binomial distribution for 10^4^ events, the significance of differences between classes was calculated using the chi-square method. Boxplots were built by RStudio version 3.5.1 (R Core Team, 2021).

## Results

### Patches Seeded With Human Endothelial and Smooth Muscle Cells From Cardiac Explants Show Normal Patency After Engraftment in the Abdominal Aorta of Mice

We tested tissue-engineered constructs with EC and SMC from cardiac explants for the repair of vascular defects. We pre-seeded *in vitro* PCL patches with endothelial cells on one side and with smooth muscle cells on the other and then implanted them in 23 SCID mice of the experimental group with induced abdominal aortic defect (Supplementary Figure S1A). The control group consisted of 12 mice with the same defect, which was implanted with PCL patches without cells. Patch implantation details are presented in the video (Supplementary Video S1). The patency of the abdominal aorta was assessed at the 4th, 12th, and 24th weeks after surgery using ultrasound and MRI.

**FIGURE 2:**
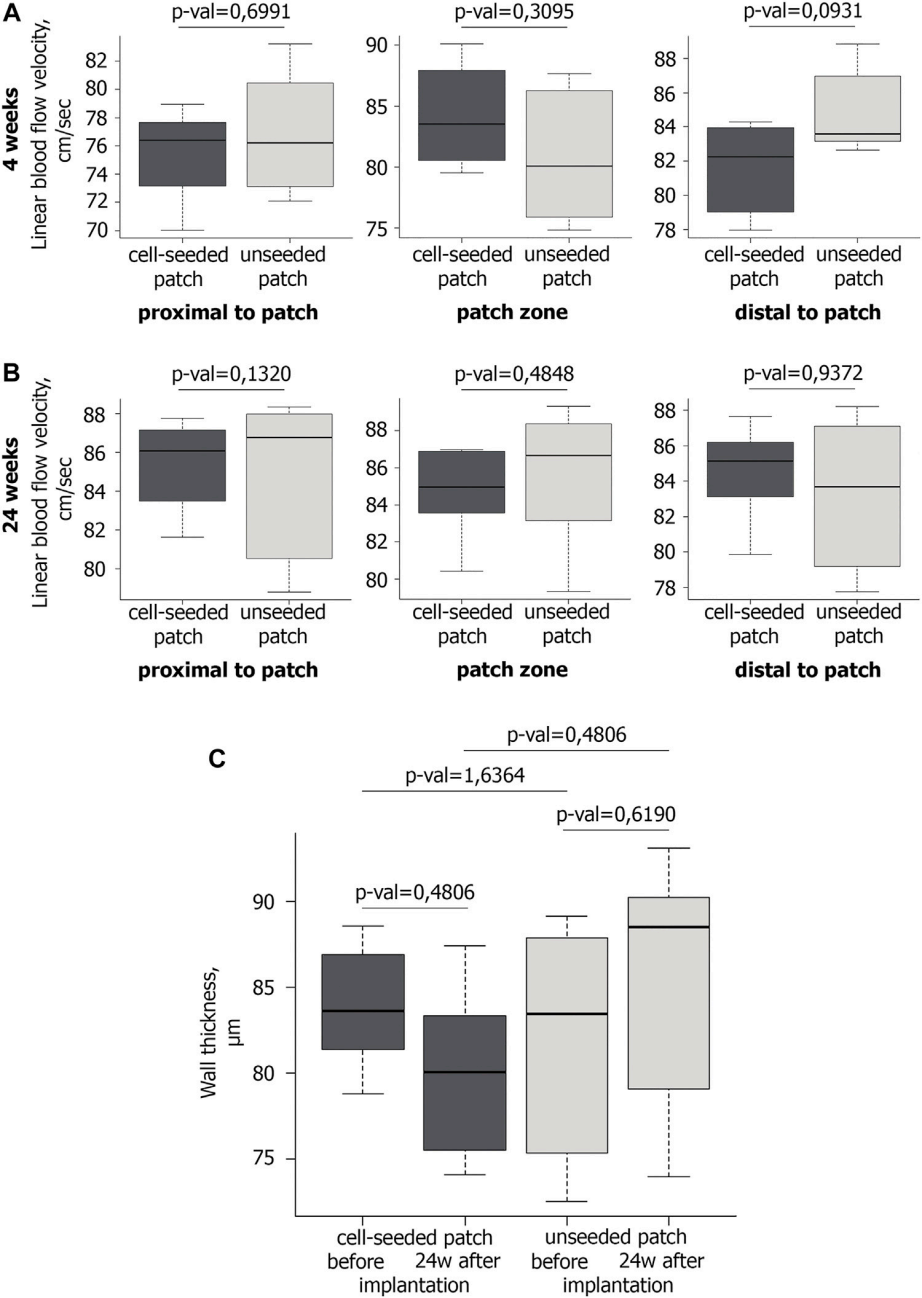
Blood flow and wall thickness in the operated mouse aorta. **A,B** Ultrasound examination of the linear blood flow velocity in the area of the implanted vascular patch and around it at the 4th **(A)** and 24th **(B)** weeks after implantation. At the 4th week after implantation n = 6 for cell-seeded patch, n = 6 for unseeded patch; at the 24th week after implantation n = 6 for cell-seeded patch, n = 3 for unseeded patch. **(C)** Wall thickness of vascular patches before (n = 6 for cell-seeded patch, n = 6 for unseeded patch) and 24 weeks after implantation (n = 6 for cell-seeded patch, n = 3 for unseeded patch).

Doppler ultrasound scanning detected no significant differences in the linear blood flow velocity (BFV) between the experimental and control groups in the patch implantation area at 4th week of observation (Figure 2A). Similarly, we have not found any statistically significant differences in BFV after 24 weeks (Figure 2B).

Ultrasound triplex study showed that in all mice of the control and experimental groups at all control points the abdominal aorta patency was preserved, the blood flow rate was within the normal range (Figures 2A,B). Maintaining normal blood flow without increasing the BFV in the patch implantation area, allow us to exclude hemodynamically significant narrowing and/or occlusion of the abdominal aorta in mice.

No significant difference in the wall thickness was identified between the groups before implantation and 24 weeks after implantation (Figure 2C).

**FIGURE 3:**
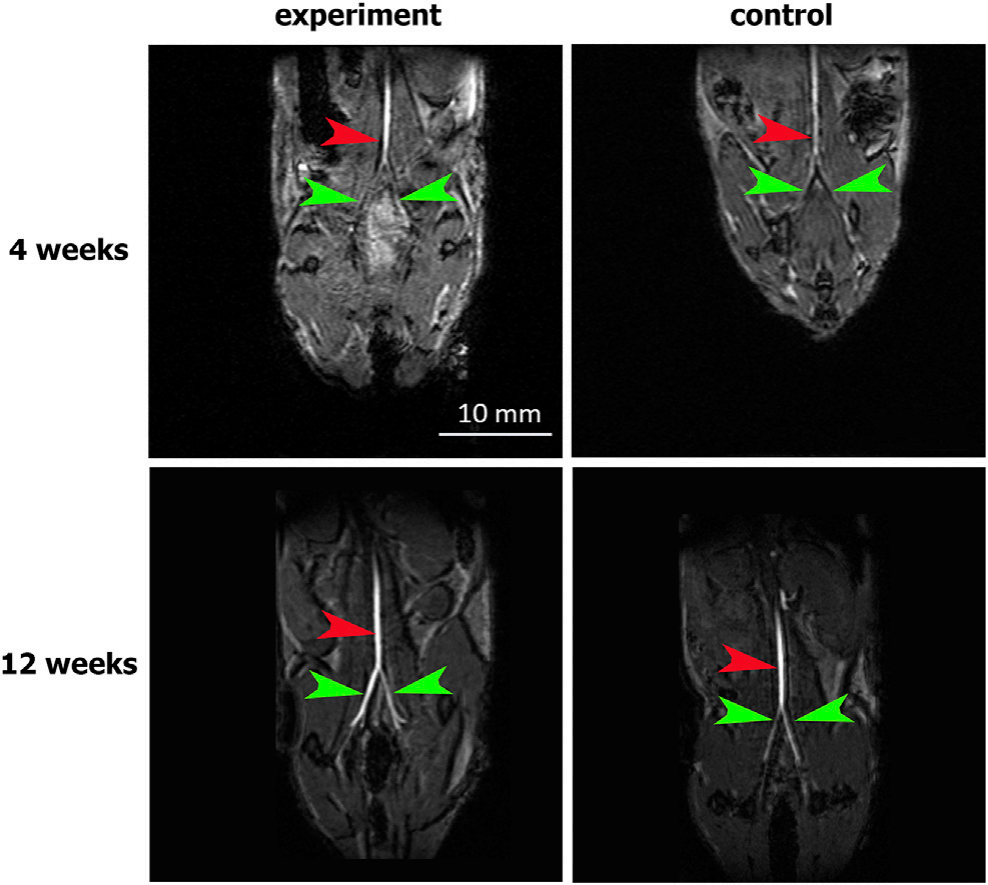
MRI assessment of aortic patency. “Experiment” and “control” are mouse groups implanted with cell-seeded and unseeded patches, respectively. N = 6 for each group at each time point. Red arrows indicate the patent abdominal aorta, green arrows indicate the patent iliac arteries.

The abdominal aorta patency 4 weeks after surgery was confirmed by abdominal cavity MRI. In both the experimental and control groups, the abdominal aorta and iliac arteries were visualized without filling defects, which excluded hemodynamically significant stenosis or occlusions of the abdominal aorta. The absence of both aneurysms and ruptured patches indicates their mechanical strength (Figure 3).

Thus, at the hemodynamic examination level over 24 weeks, we did not find any advantages between PCL patches with and without cells.

### Polycaprolactone Patches With Cells Engrafted Into the Abdominal Aorta in Mice Form the Vessel Wall Structure With Endotheliocyte Monolayer and Smooth Muscle Cell Layers

The patch was implanted in such a way that the one side, covered with endothelial cells, was in contact with the bloodstream, while the other side, seeded with smooth muscle cells, was in contact with the surrounding tissue (Supplementary Figure S1B). After the patch was explanted and dissected from the aorta, a qualitative visual observation evidenced that about 80% of the patch from the inside was in contact with blood, while ∼20% was in contact with the arterial tissue.

In order for a tissue-engineered vascular patch to be able to integrate properly and perform its functions correctly, it requires to have a monolayer of endothelial cells and layers of smooth muscle cells. In addition, adequate patch functioning requires the cells within it to produce an appropriate extracellular matrix. We analyzed how these conditions are met for PCL patches explanted from the mouse abdominal aorta at weeks 2, 4, 12, and 24 of the experiment.

**FIGURE 4:**
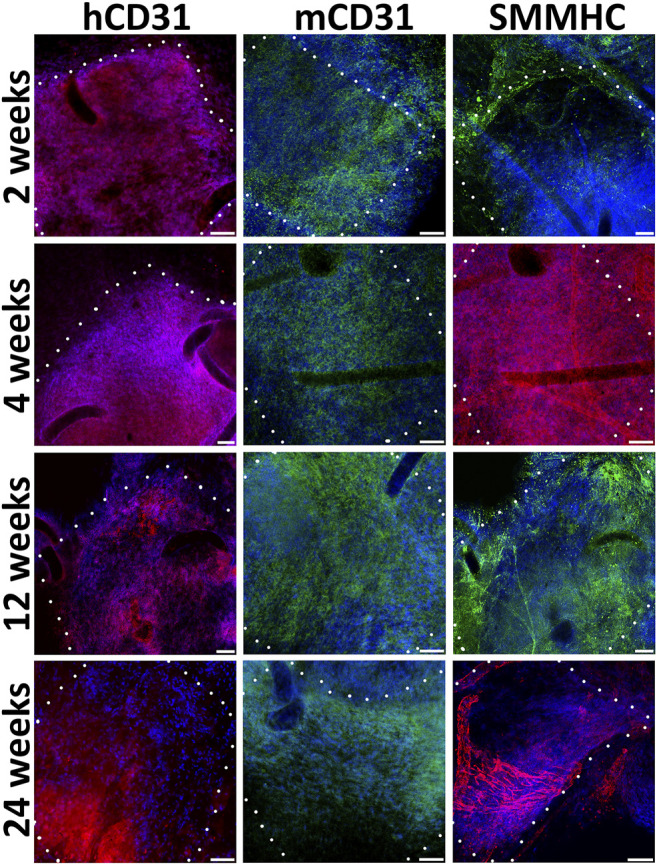
Immunofluorescent staining of cell-seeded patches with antibodies to endotheliocyte (hCD31, mCD31) and smooth muscle (SMMHC) cell markers at control points of the experiment. The borders of the patch are shown with a dashed line. *Scale bar* 50 μm.

We find out that already 2 weeks after engraftment pre-seeded PLC patches on the inner surface contain not only human endothelial cells (hCD31), but also mouse endothelial cells (mCD31) (Figure 4). Human CD31-positive cells are detected at all observation points, while from the 12th to 24th week, their number drastically decreases. The number of murine CD31-positive cells increases by 12 weeks. At week 24, mouse CD31-positive cells almost completely cover the inner surface of the pre-seeded PLC patch. At all observation points on the outside surface of pre-seeded PLC patches, we also identified smooth muscle cells positive for the smooth muscle myosin heavy chain (SMMHC) marker (Figure 4).

**FIGURE 5:**
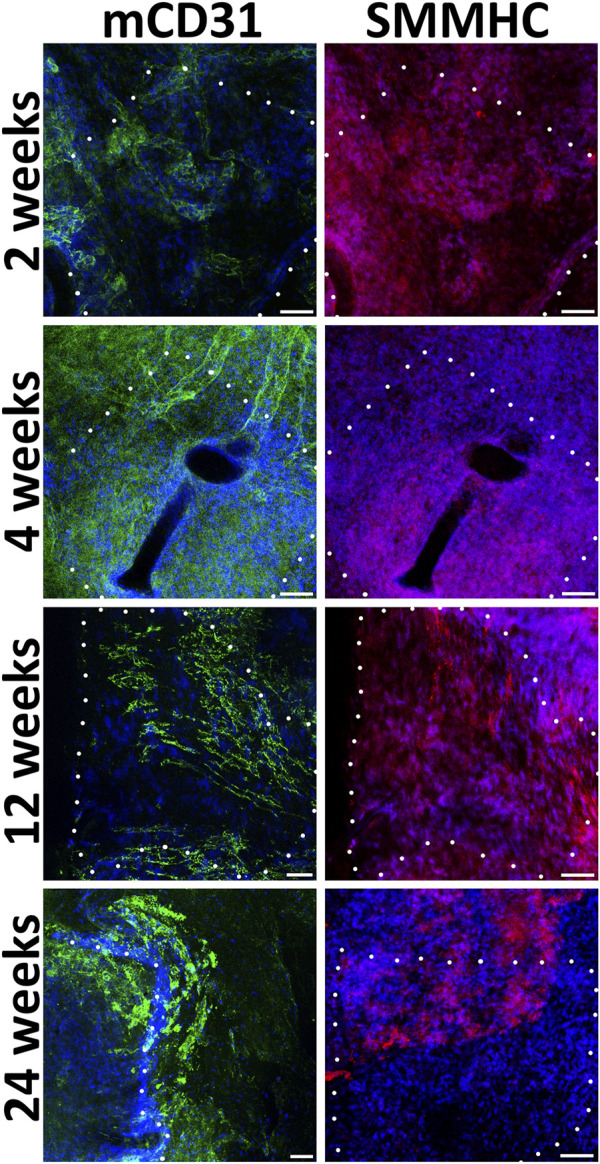
Immunofluorescent staining of unseeded patches with antibodies to endotheliocyte (mCD31) and smooth muscle (SMMHC) cell markers at control points of the experiment. The borders of the patch are shown with a dashed line. *Scale bar* 50 μm.

During implantation even after 24 weeks, non-seeded PCL patches, in contrast to pre-seeded ones, lack a uniform monolayer of mouse endothelial and smooth muscle cells (Figure 5). In the absence of human EC and SMC, the density of mouse cells on the patches was higher near the edges adjacent to the recipient tissues. Usually, on the inner patch surfaces, along with or instead of endothelial cells, we detected either SMC or cells negative for vascular markers.

**FIGURE 6:**
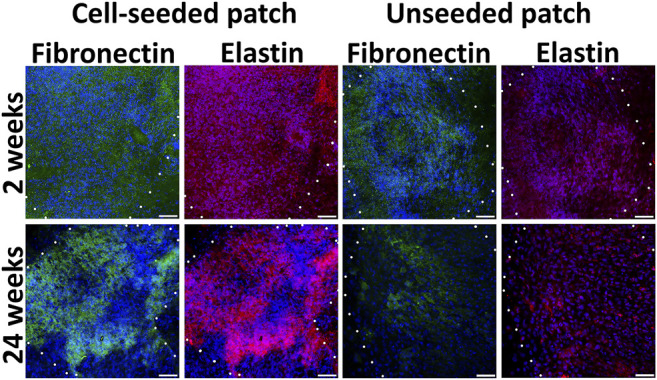
Extracellular matrix components in cell-seeded and unseeded patches. The borders of the patch are shown with a dashed line. *Scale bar* 50 μm.

Extracellular matrix components as fibronectin and elastin attributable for ECs and SMCs respectively were detected homogeneously in the pre-seeded PLC patches at the 4 and 24 weeks (Figure 6; Supplementary Figure S8). By the 24th week, in the non-seeded PLC patches, extracellular matrix attributable to vessels are accumulated weakly, only in some areas. (Figure 6). Probably, not all cells that colonized to unseeded patch are capable of producing fibronectin and elastin. We propose that a weak production of extracellular matrix on the unseeded patches may be associated with uneven colonization of the patch with both EC and SMC, leading to the absence of a continuous monolayer. The unseeded patch contains no pre-formed niches for attracting the appropriate cell types. Colonization after implantation can also occur with nonspecific cells that do not produce the matrix in such an amount as this occurs on a patch pre-seeded with cells.

**FIGURE 7:**
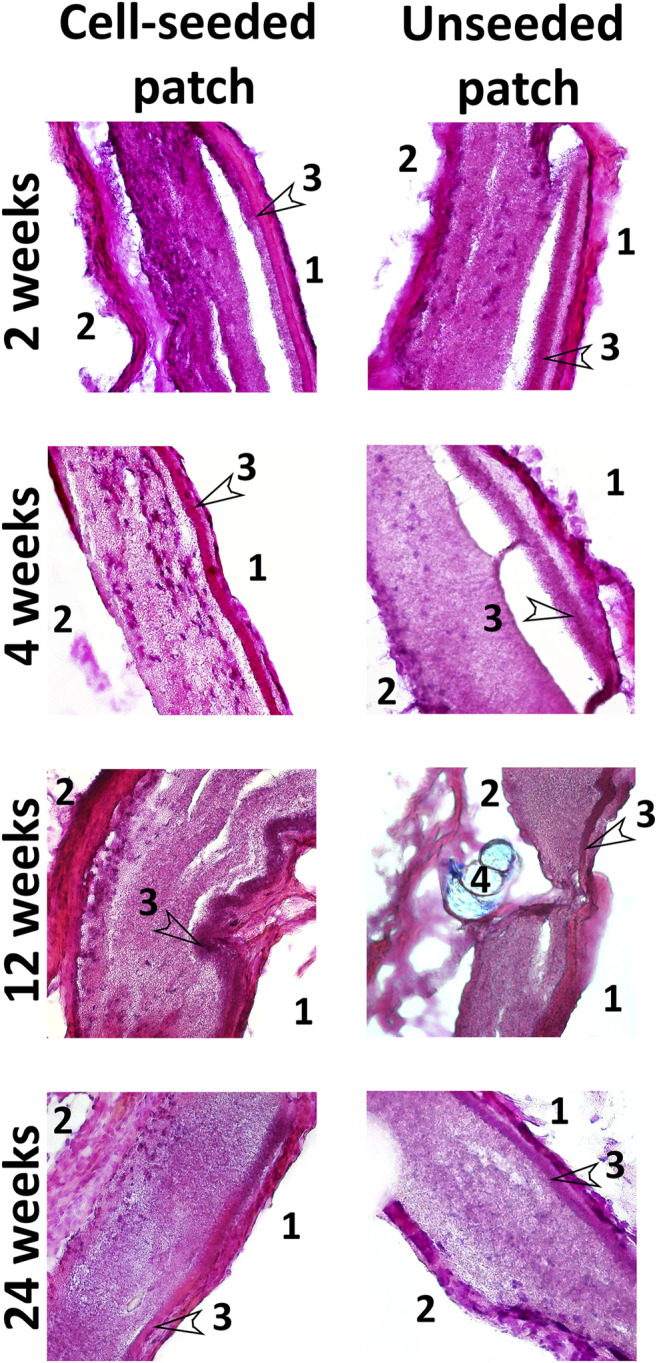
Hematoxylin and eosin staining of cryosectioned patches. Magnification ×100 and 400. 1—inner side of the patch in contact with the bloodstream; 2—the outer side of the patches in contact with the surrounding tissues; 3—low-permeability inner layer; 4—suture material (Premilene 10/0).

Histological staining with hematoxylin and eosin demonstrated that patches of the experimental group contained cell nuclei in the thickness of the PLC scaffold throughout the whole observation period at all control points (Figure 7). A monolayer of endotheliocyte-like cells was visualized throughout the experiment on the inner side of the patches that contact the bloodstream. From the second week, on the outer surface of the cell-seeded patches, we identified a fibrous capsule gradually thickening over time. At all stages of the experiment, the new capsule consists of dense fibrous tissue including a high content of smooth muscle and fibroblastic cells with microvessel formation areas.

PCL patches engrafted without cells, after explantation and staining with hematoxylin and eosin, also visualized cell nuclei in their thickness after 2 and 4 weeks. This indicates that the scaffold material does not interfere with the migration of cells inward (Figure 7). However, further histological examination showed a decrease in the number of nuclei from the 12th to the 24th week (Figure 7). Colonization of acellular vascular grafts with cells from circulating blood was described early (De Visscher et al., 2012; Sánchez et al., 2018; Smith et al., 2020).

Presumably, patches from the control group are colonized *in vivo* with blood cells, which are subsequently eliminated by apoptosis. In some non-seeded PCL patches, we noted areas with loss or even absence of polycaprolactone scaffold fibers, where both individual collagen fibers and fibrosis foci often occur. Our results suggest that in the absence of functional cells within the tissue-engineered construct, it tends to more rapid degradation, leading to a partial loss of the patch structural integrity. At all stages of the study, no intact monolayer of endotheliocyte-like cells was observed on the inner surface of non-seeded patches. In contrast to the experimental group, the external fibrous capsule in the control group is characterized by a thin layer of dense fibrous tissue with an insignificant content of fibroblast-like cells and the absence of a microvasculature (Figure 7).

The patches transplanted with pre-seeded EC and SMC after isolation from the aorta of experimental mice showed external surfaces with more pronounced local neovascularization, in contrast to the control patches, where new microvessels were less developed (Supplementary Figure S1B).

Thus, the patch pre-seeding with ECs and SMCs contributes to its integration into the recipient tissue, the formation of microvasculature, as well as the scaffold material integrity. After engraftment, cells in construct continue to produce an extracellular matrix, forming a highly specialized microenvironment that attracts corresponding recipient cells, which over time replace the transplanted cells.

### Mitomycin C Treated Endothelial and Smooth Muscle Cells do Not Differ in Viability

In order to prevent the potential risk of malignancy from proliferating cells included in the tissue-engineered construct, endothelial and smooth muscle cells were treated with mitomycin C (MMC), which arrests cell division. We performed a series of tests to confirm that cells treated with MMC remain viable and functional despite the inability to divide. In particular, we showed that MMC-treated ECs and SMCs demonstrate normal viability >90%, retain specific markers, and ability to produce extracellular matrix as well as demonstrate an angiogenic potential *in vivo*.

#### Residual Traces of Mitomycin C Are Not Detected in Treated Endothelial and Smooth Muscle Cells

We examined residual traces of MMC in endothelial and smooth muscle cells after treatment by HPLC-MS. The initial concentration of MMC in the growing medium was 10 μg/mL. As this concentration of MMC lies above the chosen calibration range (Supplementary Figure S2B) it was not included in the set of calibrants. Supplementary Figure S3, S4 show the absence of MRM signal from m/z 335 to 242 transition, referring to MMC. Thus, we have shown that in ECs and SMCs no residual traces of MMC are detected according to quantitative HPLC-MS analysis.

#### XTT-Analysis of Endothelial and Smooth Muscle Cells Proliferation

**FIGURE 8:**
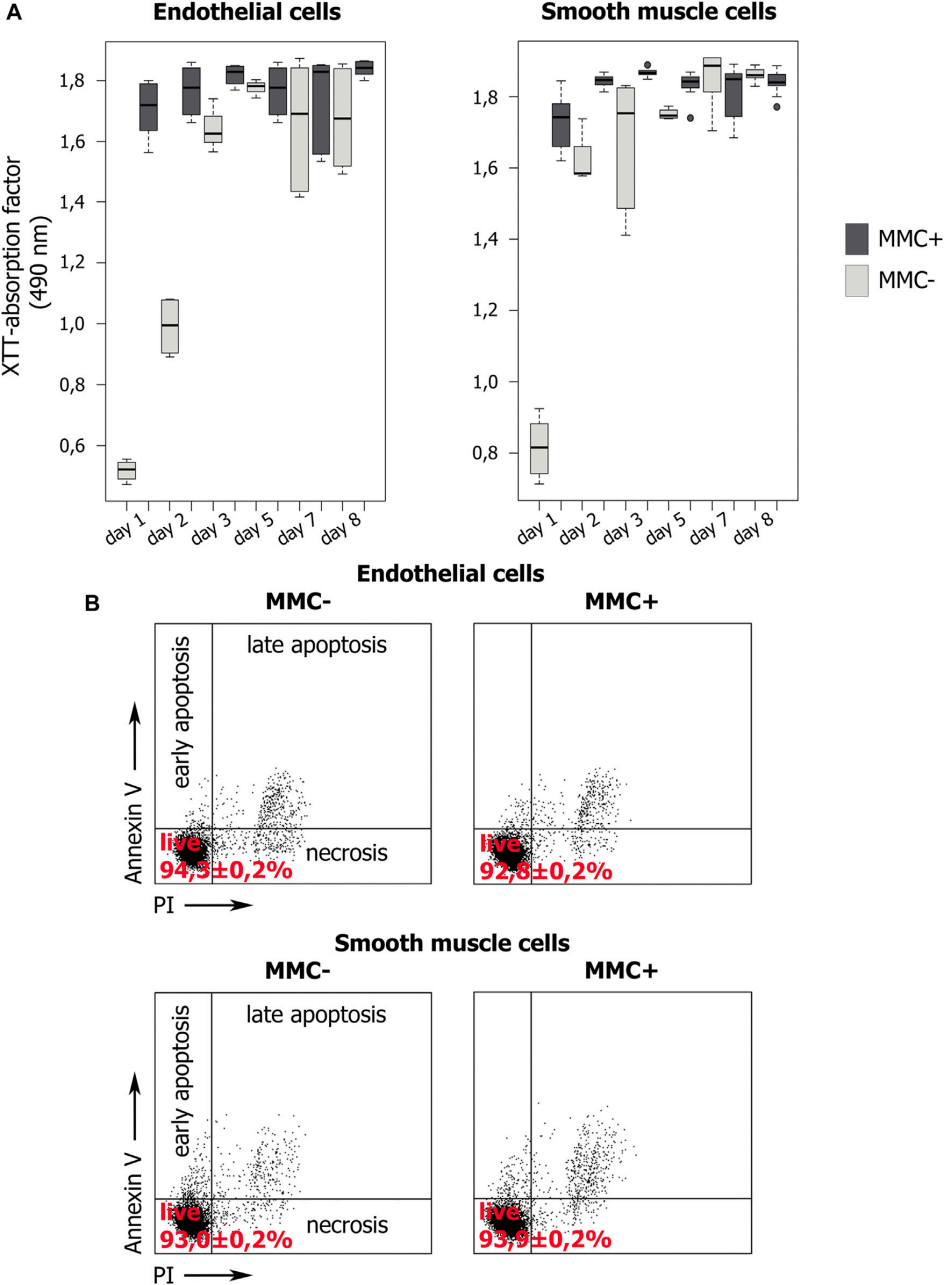
Proliferation dynamics and viability of MMC-treated (MMC+) and untreated (MMC-) endothelial and smooth muscle cells. **(A)** XTT values indirectly reflect the number of viable cells. XTT values dynamics during 8 days of cultivation demonstrate the proliferation of MMC + cells and its absence in MMC- cells. N = 9 for each group at each time point. **(B)** Percentage of viable cells stained with Propidium Iodide and Annexin V according to flow cytometry. For each group, 10^4^ events were estimated.

First, we confirmed that cells treated with MMC did stop dividing and did not increase in number within 7 days of observation, unlike untreated cells (Figure 8A). For this, we used the XTT-test, in which the XTT value reflects the number of active mitochondria. Measuring the XTT value over 8 days allows to indirectly estimate the number of cells and their proliferation.

XTT showed that endothelial and smooth muscle cells untreated with MMC actively proliferate. For both untreated EC and SMC, the highest XTT values reflecting the maximal number of viable cells are observed by day 7. Comparison for adjacent days showed no statistically significant differences (*p* > 0.05), which indicates a trend towards a gradual increase in cell number. The exception is smooth muscle cells from 5 to 7 observation days (*p* = 0.03954). Comparison of distant points (day 1 and day 7) showed significant differences for both endothelial (*p* = 0.000219) and smooth muscle (*p* = 0.036295) cells, indicating active proliferation of cells not treated with MMC.

Additionally, XTT results indicate that both types of mitotically inactivated cells do not increase their number. We did not reveal a statistically significant difference in the XTT values for endothelial cells in the period from 2 to 7 days, and for smooth muscle cells for the entire observation period from 1 to 8 days (Figure 8A). For endothelial cells treated with MMC, a significant difference was found when comparing on days 1 and 2, as well as on days 7 and 8. We believe that this is not directly related to cell division, but reflects the result of the functional state of mitochondria.

On the 8th day, in mitotically active cell populations, a decrease in viability values is observed, which may be associated with contact inhibition of proliferation due to a rapid achievement of critical density in the cell layer.

When comparing the XTT values for cells non-treated and treated with MMC a significant difference was revealed in critical points, namely on days 5 and 7 both for EC and SMC.

Thus, XTT test confirms that treated with MMC are indeed non-proliferating. Both concentration of MMC and the exposure time we used were sufficient to arrest cell division.

#### Cell Viability Assay

We examined the viability of endothelial and smooth muscle cells using cells staining with propidium iodide (PI) and annexin V (Annexin V) followed by flow cytometry (Figure 8B). A cell population negative for both markers is viable. We compared non-treated and MMC-treated ECs and SMCs. In all cases, the cells demonstrate normal viability >90% without statistically significant differences in the percentage of viable cells (*p* > 0.01). Thus, the used MMC concentration and exposure time do not reduce the viability of the treated cells.

In addition, we analyzed the viability of MMC-treated cells after cultivation for 5 days on a PCL patch using TMRM, which stains mitochondria in living cells, and FITC-conjugated Annexin V, which detects apoptosis (Supplementary Figure S5). The number of viable TMRM-positive cells when counted in 10 fields of view varied from 94.3 to 97.1% (the number of cells counted is about 2,000 for each sample).

### Karyotyping of Cells

Endothelial and smooth muscle cells untreated with MMS have a normal female karyotype and show no visible aberrations after examination of routine G-banding (Supplementary Figure S6A,B). Molecular karyotyping revealed 18 CNVs (microdeletions and microduplications) ranging from 50 to 230 kb (Supplementary Table S1), occurring from one to 11 times among 11 analyzed samples (EC MMC +, n = 3; EC MMC-, n = 3; SMC MMC +, n = 3; SMC MMC-, n = 2). We found 7 out of 18 CNVs in only MMC-untreated cells, 5 CNVs in only MMC-treated cells, and 6 CNVs in both MMC-untreated and treated cells (Supplementary Figure S6C). We showed that the number of CNVs did not significantly differ in the groups of treated and untreated cells (Supplementary Figure S6D). We also found no differences when comparing samples within endothelial or smooth muscle cells.

### Endothelial and Smooth Muscle Cells Treated with Mitomycin C Retain Specific Markers and the Ability to Produce Extracellular Matrix

**FIGURE 9:**
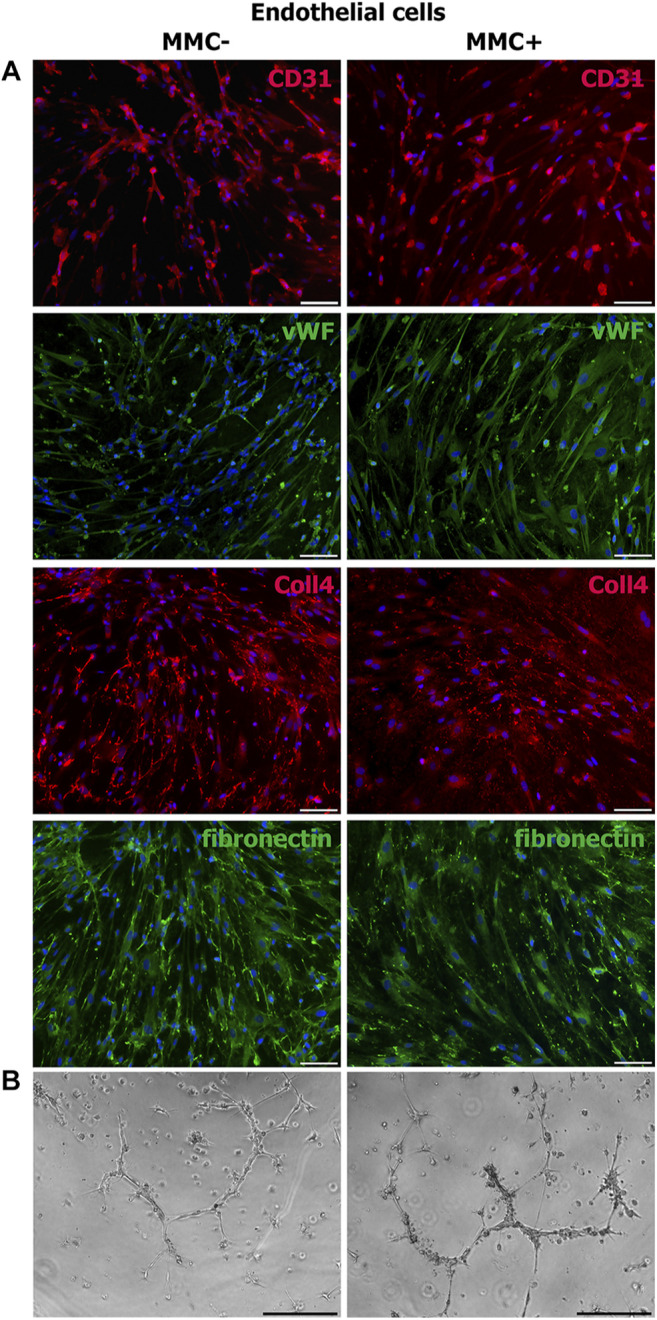
Properties of MMC-treated (MMC+) and untreated (MMC-) endothelial cells. **(A)** MMC + endothelial cells reveal CD31^−^and vWF-positive staining and produce extracellular matrix (collagen IV- and fibronectin-positive staining). **(B)** MMC+ and MMC- endothelial cells show no difference in the ability to form capillary-like structures in Matrigel. *Scale bar* 100 μm.

Using immunofluorescence staining, we assessed cell-specific properties in endothelial and smooth muscle cells treated with MMC. Endothelial cells treated with MMC demonstrate endothelial markers CD31 and von Willebrand factor, as well as produce extracellular matrix components type IV collagen and fibronectin (Figure 9A), retain the specific ability to form capillary-like 3D structures within matrigel layer (Figure 9B).

**FIGURE 10:**
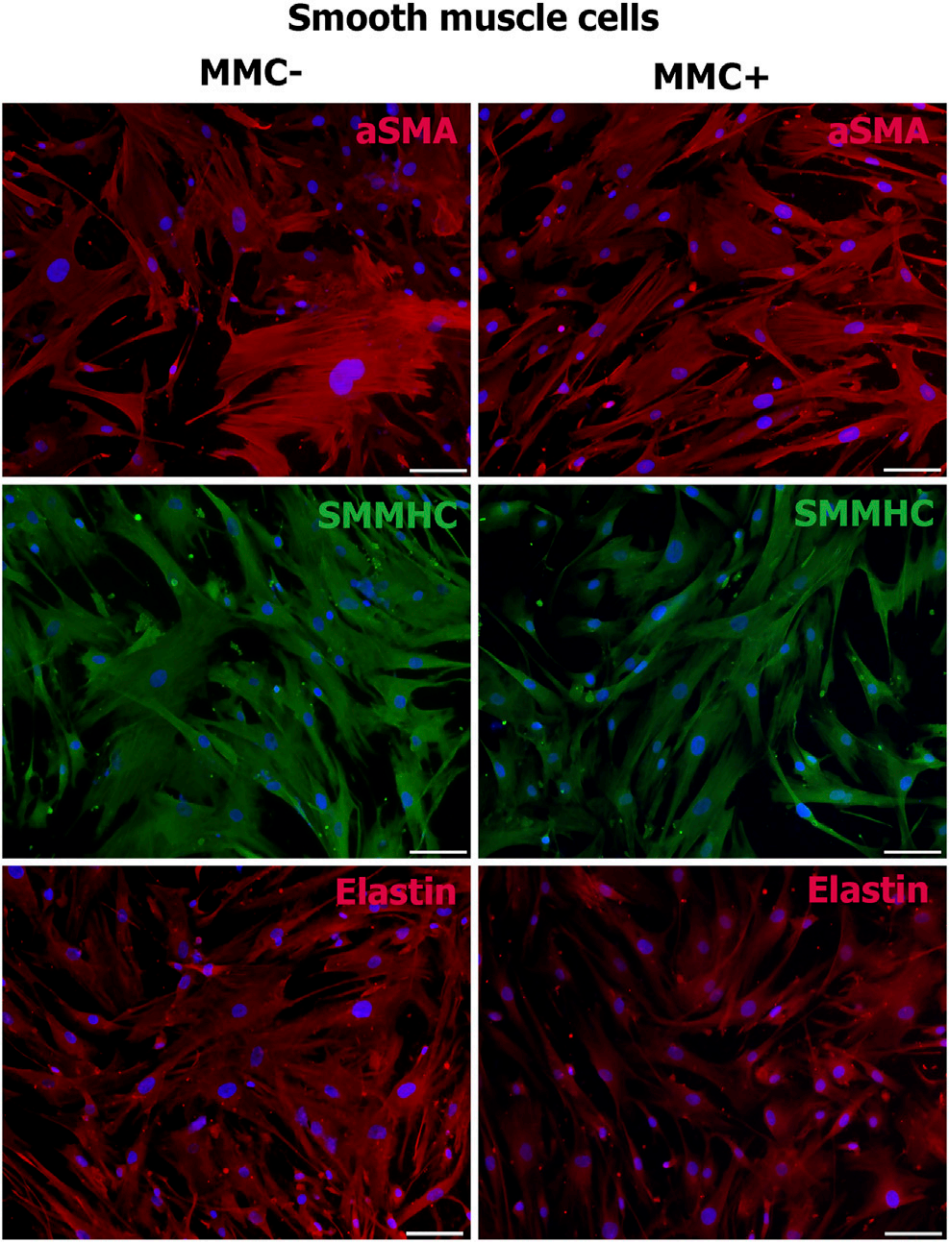
MMC-treated (MMC+) and untreated (MMC-) smooth muscle reveal αSMA- and SMMHC-positive staining and produce extracellular matrix elastin. *Scale bar* 100 μm.

Smooth muscle cells treated with MMC showed properties attributable to normal SMCs as α-smooth muscle actin, SMMHC, and extracellular elastin production (Figure 10).

Thus, endothelial and smooth muscle cells treated with MMC do not lose specific markers and are able to accumulate extracellular matrix. Mitotically inactivated endothelial cells retain the ability to form capillary-like structures *in vitro*.

### Untreated and Mitomycin C Treated Endothelial and Smooth Muscle Cells Show No Difference in Angiogenic Potential

An important step in assessing the morphofunctional properties of endothelial and smooth muscle cells is to test their ability to induce angiogenesis *in vivo*. The standard test for angiogenic potential includes injecting cells within matrigel into the abdominal region of immunodeficient SCID mice. The angiogenic effect of the tested cells is due to the paracrine effect, i.e. angiogenic cytokines and growth factors secreted by cells and attracting the recipient’s cells (Yamahara et al., 2008; Yamahara and Itoh, 2009; Rufaihah et al., 2013). Angiogenic potential is assessed based on total length, branching, and other vasculature parameters in cryosections 14 days after injection. In our study, we compared the ability to stimulate neovascularization between actively dividing and mitotically inactivated cells in order to prove the functionality and potential suitability of ECs and SMCs treated with MMC for regenerative purposes. We injected a 1:1 mix of endothelial and smooth muscle cells since it is known that together they provide more pronounced angiogenesis when quantifying the total length and branching of capillaries (Yamahara and Itoh, 2009).

**FIGURE 11:**
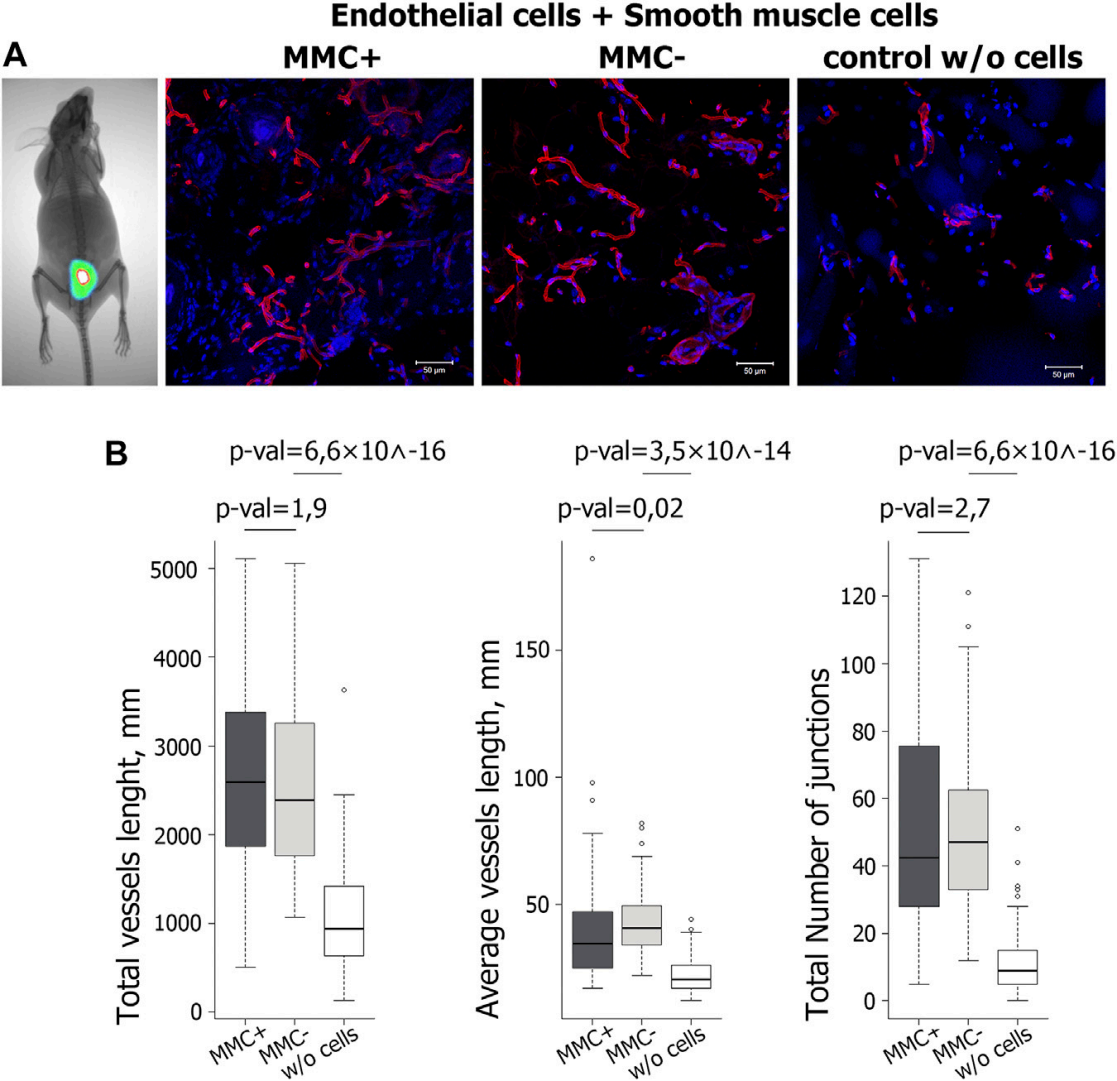
Evaluation of functional properties of MMC + cells *in vivo*. **(A)** Visualization of the injected mix (Matrigel and cells stained with the vital dye MitoTracker Deep Red FM) after 14 days with a Kodak *In-Vivo* Multispectral Imaging System device. N = 2 (on the left). Vasculature in cryosections of the Matrigel plug at day 14 after injection of Matrigel & MMC + endothelial and smooth muscle cells, Matrigel & MMC- endothelial and smooth muscle cells, and Matrigel only (control w/o cells) is detected by isolectin B4 Alexa 594 conjugate staining (red). N = 5 for each group. *Scale bar* 100 μm. **(B)** Diagrams representing the quantitative parameters of vessels positive for isolectin B4 Alexa 594 conjugate staining, imaged on a fluorescent microscope in 10 random fields of view.

To show that throughout the experiment, the injected cells reside in the implantation area, we injected two mice with a mixture of endothelial and smooth muscle cells stained by the intravital mitochondrial dye MitoTracker Deep Red FM. We visualized viable fluorescently labeled cells in the injection area on the 14th day of the experiment (Figure 11A). Matrigel without cells was injected as a control.

Quantitative analysis of vasculature in cryosections 14 days after injection showed that the total vessel length was significantly higher in implants including matrigel with cells compared to matrigel only. At the same time, no significant difference was found between the groups that received the cells treated and untreated with MMC (Figure 11B).

Thus, ECs and SMCs after mitotic inactivation do not decrease their angiogenic potential *in vivo*. The combined injection of endothelial and smooth muscle cells increases the number of vascular structures by 2–3 times compared to the injection of matrigel without cells.

### Mitomycin C Treated Endothelial and Smooth Muscle Cells Have No Tumorigenic Effect

The tumorigenic effect from the mitotically inactivated endothelial and smooth muscle cells was evaluated on immunodeficient SCID mice. The experiment was carried out on 6 groups of mice injected with:

group 1. Endothelial cells untreated with MMC, 5 mice; group 2. Smooth muscle cells untreated with MMC, 4mice; group 3. Endothelial cells treated with MMC, 5 mice; group 4. MMC-treated smooth muscle cells, 4 mice; group 5. Control—matrigel without cells, 5 mice; group 6. Highly tumorigenic control—HEK 293FT cells, 5 mice.

The duration of the experiment in groups 1–5 was 12 weeks, in group 6 it was 19–21 days. The shortened experiment period in the highly tumorigenic control group is explained by the rapid formation of a significant tumor (Supplementary Figure S7).

**FIGURE 12:**
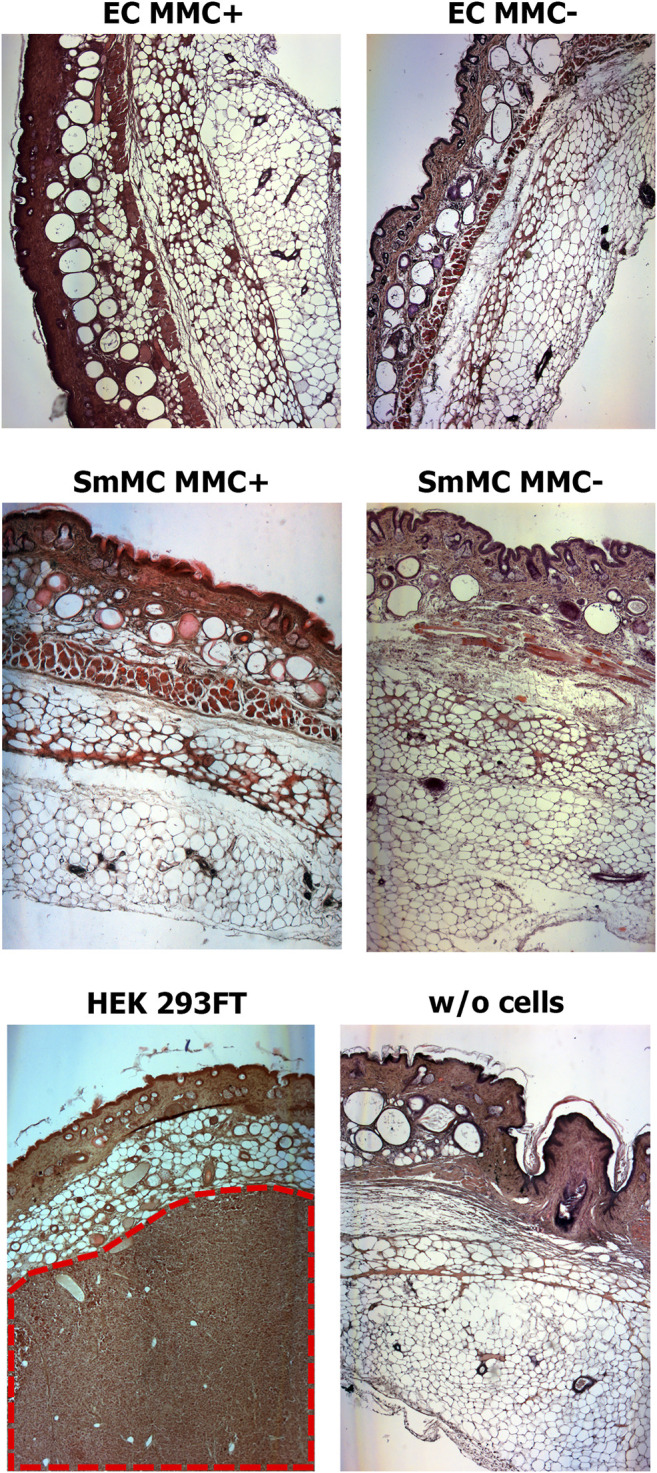
Mitotically inactivated (MMC+) endothelial (EC) and smooth muscle (SMC) cells show no tumorigenic effect *in vivo*. Histological sections show the absence of a tumorigenic effect 12 weeks after injection of Matrigel with MMC+ (n = 5 for MMC + EC, n = 4 for MMC + SMC) or MMC- cells (n = 5 for MMC- EC, n = 4 for MMC- SMC) or Matrigel only (w/o cells; n = 5). The tumor (shown in red dotted line) is detected only after injection of the highly-tumorigenic HEK293FT cell line (n = 5). *Magnification*—50X.

Analysis of histological sections from the cell injection area showed the absence of any malignization in the groups of endothelial and smooth muscle cells treated and untreated with MMC, as well as in the control group of Matrigel without cells (Figure 12). In the group injected with HEK 293FT cells, tumorigenic foci were found at the injection site (Figure 12). Thus, EC and SMCs from cardiac explants appeared to be low- or non-tumorigenic *per se*, while mitotic inactivation seems to take away the remaining chance of malignization.

## Discussion

Human heart material is one of the promising sources for regenerative medicine. More than 20 years ago, after transplantation, human endothelial cells were derived from the recipient’s whole heart by the reperfusion technique (Gräfe et al., 1994; McDouall et al., 1996). Cells from this source found no use for regenerative medicine while becoming applicable in pathophysiological and pharmacological cardiovascular studies.

Postoperative atrial tissue is widely used to obtain various cell types. In 2017 (Monsanto et al., 2017), after implantation of a left ventricular assist device, postoperative tissue samples were used to obtain three cell lines, namely cardiac progenitor cells, endothelial progenitor cells, and mesenchymal stem cells. In our previous study in 2017, we derived and cultivated non-progenitor mature endothelial and smooth muscle cells from surgical discarded right atrial appendage and right ventricular myocardium (Zakharova et al., 2017).

In this study, we showed that endothelial and smooth muscle cells derived from cardiac explants and seeded on a tissue-engineered construct improve vascular graft properties in the postimplantation period in an immunodeficient mice model. Implantation to SCID mice was used in many works as the first stage for testing cell-contained tissue-engineered constructs (Mirensky et al., 2009; Tara et al., 2014; Tara et al., 2015). The synthetic material polycaprolactone was our choice as scaffold backbone because it is a widely used biodegradable material for the manufacture of vascular scaffolds due to its successful clinical use (Tara et al., 2014). In addition, small diameter tissue-engineered vessels made from PCL show favorable biocompatibility in large animal models (Fang et al., 2021).

We propose new approaches to solving three problems in the development of tissue-engineered vascular substitutes. First, to fabricate a cell-containing tissue-engineered construct, we suggest using as a cell source autologous ECs and SMCs isolated and expanded from the waste postoperative material of patients. These cells can be cryopreserved until reoperation is required. These cells could also be used as allogeneic, for example, when the donor and recipient HLA genotypes match. Secondly, we find that preliminary seeding of cells on a synthetic scaffold improves its engraftment as well as postimplantation characteristics, bringing it closer to a natural vessel. Third, to overcome the potential tumorigenic effect of dividing cells populated on a scaffold, we propose to stop their mitotic division using MMC.

Tumorigenic risk is one of the most important aspects to consider when using cell products based on differentiated derivatives of induced pluripotent stem cells (Sato et al., 2019; Wuputra et al., 2020), mesenchymal stromal cells, and their derivatives (Meier et al., 2013; Li et al., 2021), as well as on hematopoietic cells (Heydari et al., 2020). Vascular cells are capable of malignancy, although rarely. Known cases of endothelial malignancy are very aggressive tumors with drug resistance, a high tendency to relapse, and metastases, such as endovascular papillary hemangioendothelioma, angiosarcomas, Kaposi’s sarcoma (Ravi and Patel, 2013; Wagner et al., 2017). *In vitro* cell cultivation, used in tissue engineering approaches, is associated with the phenomenon of spontaneous cell immortalization and overcoming the Hayflick limit, which directly leads to cell malignization (Forsyth et al., 2004; Rea et al., 2006; Jiang, 2010; Schmedt et al., 2012). As a result, cells with aberrant telomerase expression and unlimited division are preferably selected in heterogeneous primary cell culture. Thus, the development of approaches that control/arrest cell division is essential for cells used in regenerative medicine.

The cells from the waste postoperative material of cardiac explants seem to be preferable for autologous transplantation since it avoids the immunological problem with graft rejection. However, autologous cells can only be used for re-operation. Nevertheless, autologous cells are not available to all patients at the required time, and it takes some time to obtain them in any sufficient amount, thus, the potential use of these cells for allogeneic transplantation should be considered. In this case, donor cells obtained beforehand can be used as an allogeneic source for tissue engineering. To suppress the recipient’s immune response against transplanted allogeneic cells, two principal approaches are possible, namely HLA-typing and immunosuppression (Petrus-Reurer et al., 2021). HLA typing allows the transplantation of donor cells as an allogeneic source for tissue engineering if they match the recipient’s HLA genotype. Long-term use of immunosuppression has a number of potential limitations associated with serious toxic side effects such as the increased risk of infection, cancer, and organ damage (e.g., renal failure) (Karantalis et al., 2015; Cooke et al., 2017; Petrus-Reurer et al., 2021). However, since the cells seeded on a vascular graft are necessary to attract the cells of the recipient and then are eliminated, we can assume that the duration of immunosuppression can be not long. In addition, several studies showed that treating cells with MMC suppresses the T-cell response and does not require additional immune suppression when these cells are used for allotransplantation (Jiga et al., 2007; Gunji et al., 2008; Terness et al., 2009). However, more detailed studies are necessary to select an appropriate approach to prevent an allogeneic cell rejection reaction.

Our results suggest that E and SM cells from cardiac explants are not only useful for seeding on small diameter tubular grafts but are also valuable for vascular patch design.

In our work, we did not analyze the calcification of the tissue-engineered construct. We mainly tested a new source of cells for its fabrication and use in *vivo* models, not the scaffold material. These cells can be used with other types of synthetic scaffolds, and the composition of the scaffold more significantly contributes to calcification. Our tissue-engineered construct is a patch that repairs vessel defects, rather than a cylindrical tube. Meanwhile, the patency for engrafted construct differs depending on the length necessary for clinical bypass procedures (Skovrind et al., 2019). In this regard, we believe that further detailed studies, including calcification, should be carried out on a specific tissue-engineered vessel with the final versions of the synthetic scaffold, whereas our tissue-engineered construct is not a complete one.

Vascular patches are commonly applied in tissue repair and reconstruction in congenital cardiac surgery, including pulmonary arterioplasty, right ventricular outflow tract patching, valve leaflet augmentation, veins as well as unroofed coronary sinus repair (Gao et al., 2019; Lu et al., 2020). Vascular patches are also used for defect correction in endarterectomy surgery to restore carotid artery patency (Orrapin et al., 2021). The vascular patch provides mechanical support and is perfect for tissue regeneration. An ideal source for patching in vascular surgery is an autologous vessel. However, for many patients, this is unavailable due to medical reasons. For these patients, patch scaffolds are made from bovine pericardium or synthetic material such as polytetrafluoroethylene (PTFE), Dacron, polyurethane, and polyester (Sevostyanova et al., 2019). However, patches available today often lead to complications and are unable to completely meet the vascular surgery needs (Harrison et al., 2014). In this regard, the development of tissue-engineered vascular patches seems currently promising. Cell-containing patches may have growth potential and may be used in pediatric practice (Lu et al., 2020). A scaffold of tissue-engineered patches may be based on synthetic materials or cattle vessels including those that decellularized or seeded with autologous cells. Preclinical and clinical trials of tissue-engineered patches demonstrate patency due to neoendothelization and neomedia formation (Sevostyanova et al., 2019; Gao et al., 2019; Lu et al., 2020; Shin’oka et al., 2005). To reduce complication risks both in the early and late postoperative periods, an important issue is choosing cells from available sources and in an amount sufficient for seeding on patches.

We have shown the absence of the tumorigenic effect of endothelial and smooth muscle cells from human cardiac explants. Moreover, to avoid the potential tumorigenic effect of dividing cells, we arrested cell division with MMC. Inactivation of mitotic division allows overcoming the danger of dividing cells after transplanting cell products, because, being introduced into the patient’s body, dividing cells may give tumors.

MMC is an alkylating agent used in cancer therapy that tightly binds to individual regions of DNA, cross-links double-helical strands, inhibits DNA synthesis, and therefore suppresses cell proliferation (Terness et al., 2008). The use of MMC at non-toxic doses stops the division of cells but retains their functional characteristics (Gunji et al., 2008; Terness et al., 2008).

MMC is a clinically approved drug. In non-toxic concentrations, it is widely used in the development of regenerative approaches for medicine. In particular, based on dendritic cells treated with MMC, vaccines are being developed to combat autoimmune diseases (Terness et al., 2008). MMC is used as a potential tool for achieving donor tolerance in clinical transplantation. In animal models, donor dendritic cells treated with MMC prior to injection to the recipient block the T-cell response and prolong the survival of the allograft without additional immune suppression (Jiga et al., 2007; Gunji et al., 2008; Terness et al., 2009).

MMC is used in experimental cell therapy for Parkinson’s disease to prevent cell division that may have carcinogenic potential (Acquarone et al., 2015; Hiller et al., 2021). Recently, a method was developed for the directed differentiation of iPSCs into dopaminergic neurons using MMC to reduce the number of proliferating cells in differentiating postmitotic neurons (Hiller et al., 2021). It should be noted that, unlike other tested cytostatics as cytosine arabinoside (Ara-C) and amiodarone, MMC led to the maximum removal of proliferating cells from the population, without affecting the output of postmitotic neurons and their functioning *in vitro* and *in vivo* upon transplantation into model animals. In addition, MMC is used to prevent the division of mouse embryonic and human fibroblasts, which are feeder cells in the cultivation of ESCs and iPSCs (Michalska, 2007; Unger et al., 2008).

In our study, we showed that endothelial and smooth muscle cells from cardiac explants, when pre-plated onto vascular patches, during implantation markedly improve the construct engraftment and the scaffold material integrity, support appropriate extracellular matrix, and are eventually replaced by the corresponding vascular cells of the recipient.

We also find that treatment of endothelial and smooth muscle cells with MMC not only stops cell division and prevents the risk of potential malignancy, but also does not lead to a loss of cell viability and functional properties. Endothelial and smooth muscle cells treated with MMC retain the ability to accumulate the extracellular matrix and demonstrate angiogenic potential *in vitro* and *in vivo*.

Although it was previously reported that MMC traces remain in human and mouse embryonic fibroblasts (Zhou et al., 2015), we found no residual MMС in treated endothelial and smooth muscle cells. We suppose that our MMC-treating method enables more complete MMC elimination from treated cells. We do not add MMC directly to the growing cells. We detach cells with the enzyme and incubate the cell pellet in the growth medium with MMC, then thoroughly wash the cells pellet with PBS, and inoculate MMC-treated cells on a new culture surface, where the cells grow in a fresh medium for 5 days.

In addition, we showed no significant differences in the number of CNVs in treated and untreated MMC cells. Obviously, CNVs common to ECs and SMCs were from the original cardiac explant cells, while some CNVs may have appeared as a result of passaging of the original cell cultures. However, we obtained no evidence that MMC treatment correlates with an increase in CNVs in the cell genome. Chromosomal instability was previously reported when MMC cells were treated for 24 and 48 h (Zhou et al., 2015). Moreover, chromosomal aberrations occur in cells treated with a lower dose of MMC, in which some cells retain the ability to divide, giving subsequent generations a variety of the aberrant chromosomes. In our work, we treated cells only for 2 h and used a low-toxic concentration of MMS (10 μg/ml), at which the cells completely stop dividing.

Nevertheless, further research is needed to convert mitotically inactivated endothelial and smooth muscle cells isolated from postoperative cardiac material into medical products ready for clinical trials. We suggest that MMC treatment should be performed immediately prior to seeding endothelial and smooth muscle cells on the patch in order to exclude the MMC sorption by the patch synthetic material. After washing, cells should be enzymatically disaggregated, plated onto a synthetic scaffold, and allowed to develop extracellular matrix components for 5 days *in vitro* prior to implantation.

We believe that our proposed approach for designing tissue-engineered constructs based on mitotically inactivated patient-specific or HLA-matching allogeneic cells from waste postoperative material will be of interest for the development of safe medical cell-containing transplants.

## Data Availability

The original contributions presented in the study are included in the article/Supplementary Material, further inquiries can be directed to the corresponding author.

## Abbreviations

α-SMA, α-Smooth muscle actin; BFV, cells blood flow velocity; BSA, bovine serum albumin; CNV, copy number variation; EC, endothelial cells; ESCs, embryonic stem cells; FA, formaldehyde; FOV, fields of view; iPSCs, induced pluripotent stem cells; MRI, magnetic resonance imaging; MMC, mitomycin C; PBS, phosphate-buffered saline; PCL, polycaprolactone; PI, propidium iodide; PTFE, polytetrafluoroethylene; SCID, severe combined immunodeficient; SMC, smooth muscle cells; SMMHC, smooth muscle myosin heavy chain; TEVG, tissue-engineered vascular graft; XTT, (2,3-Bis-(2-Methoxy-4-Nitro-5-Sulfophenyl)-2H-Tetrazolium-5-Carboxanilide).
