# Supplementary figures and images for: Mitomycin-Treated Endothelial and Smooth Muscle Cells Suitable for Safe Tissue Engineering Approaches
Source: Front Bioeng Biotechnol. 2022 Mar 11;10:772981. doi: 10.3389/fbioe.2022.772981 (PMC8963790; doi:10.3389/fbioe.2022.772981)

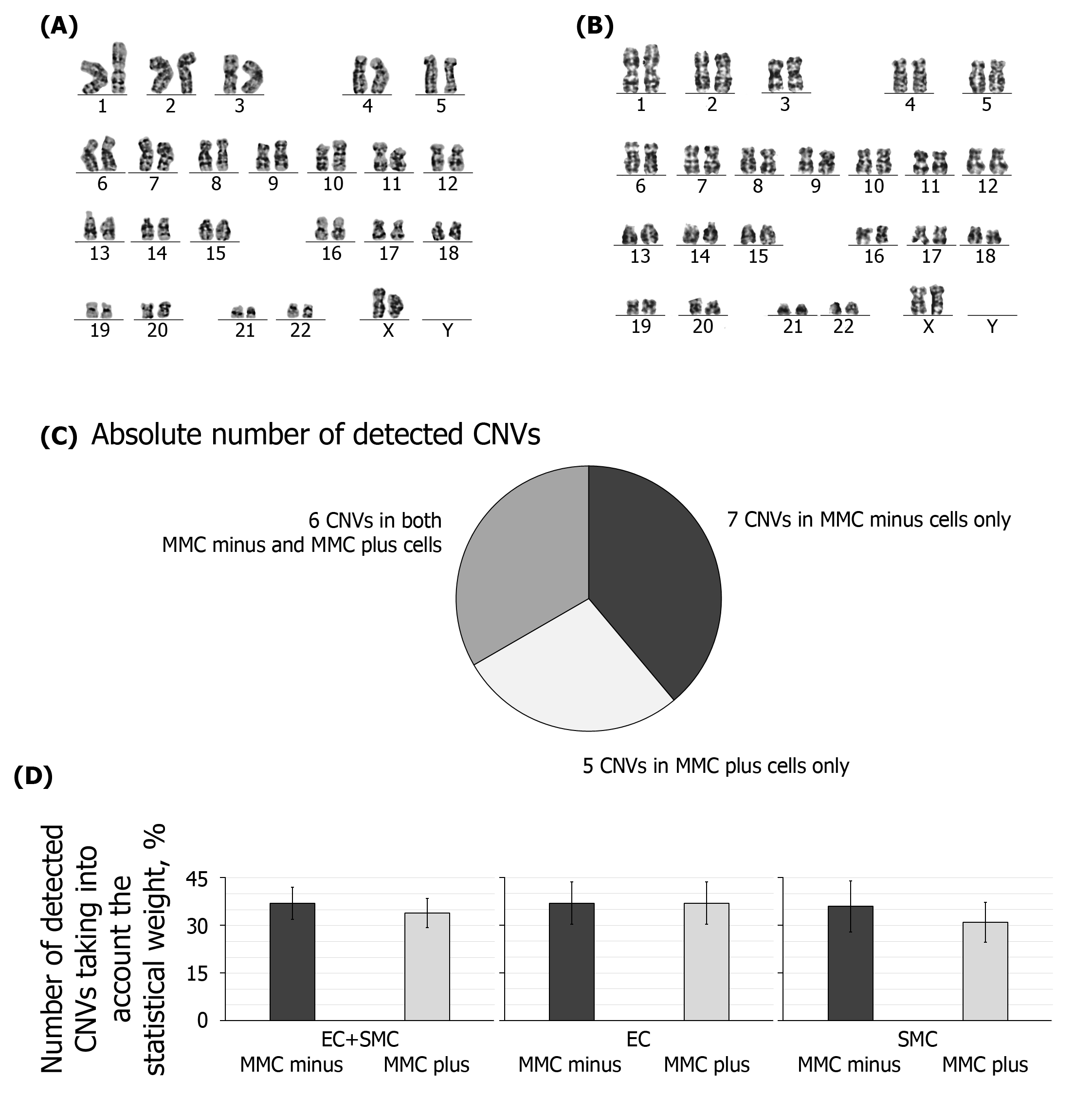

Supplement: Supplementary file 2 [file Image6.TIF]

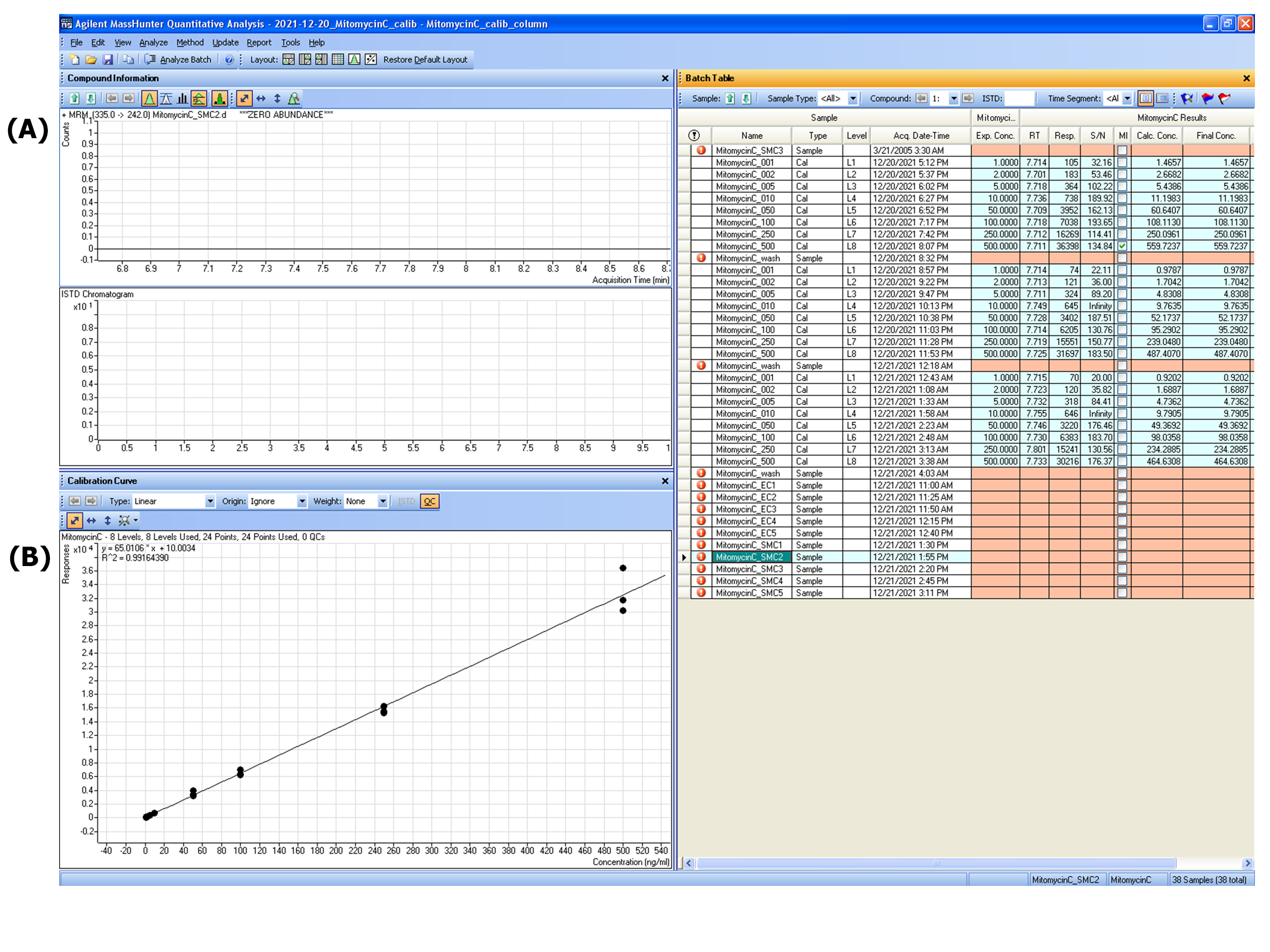

Supplement: Supplementary file 3 [file Image3.TIF]

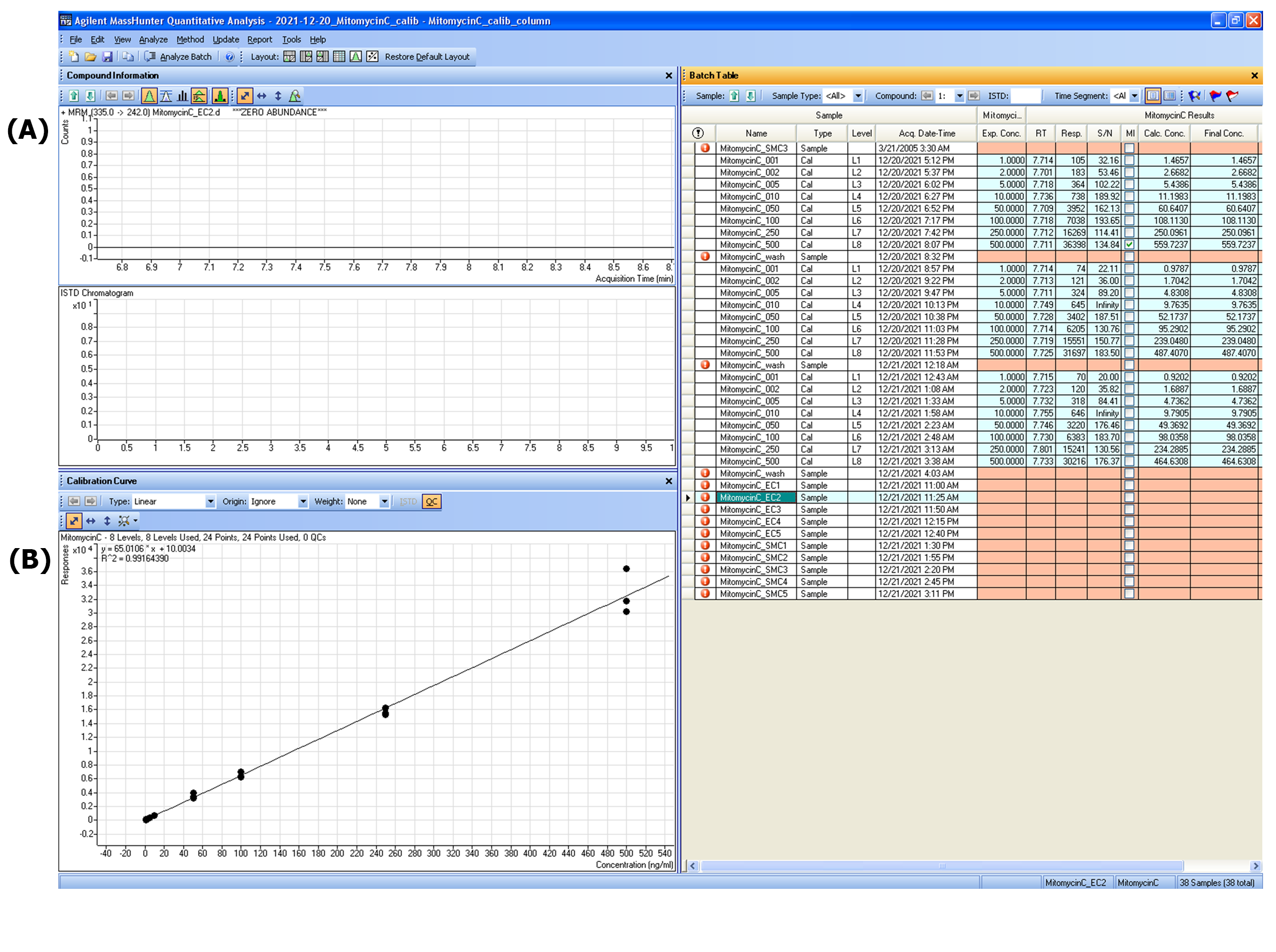

Supplement: Supplementary file 4 [file Image4.TIF]

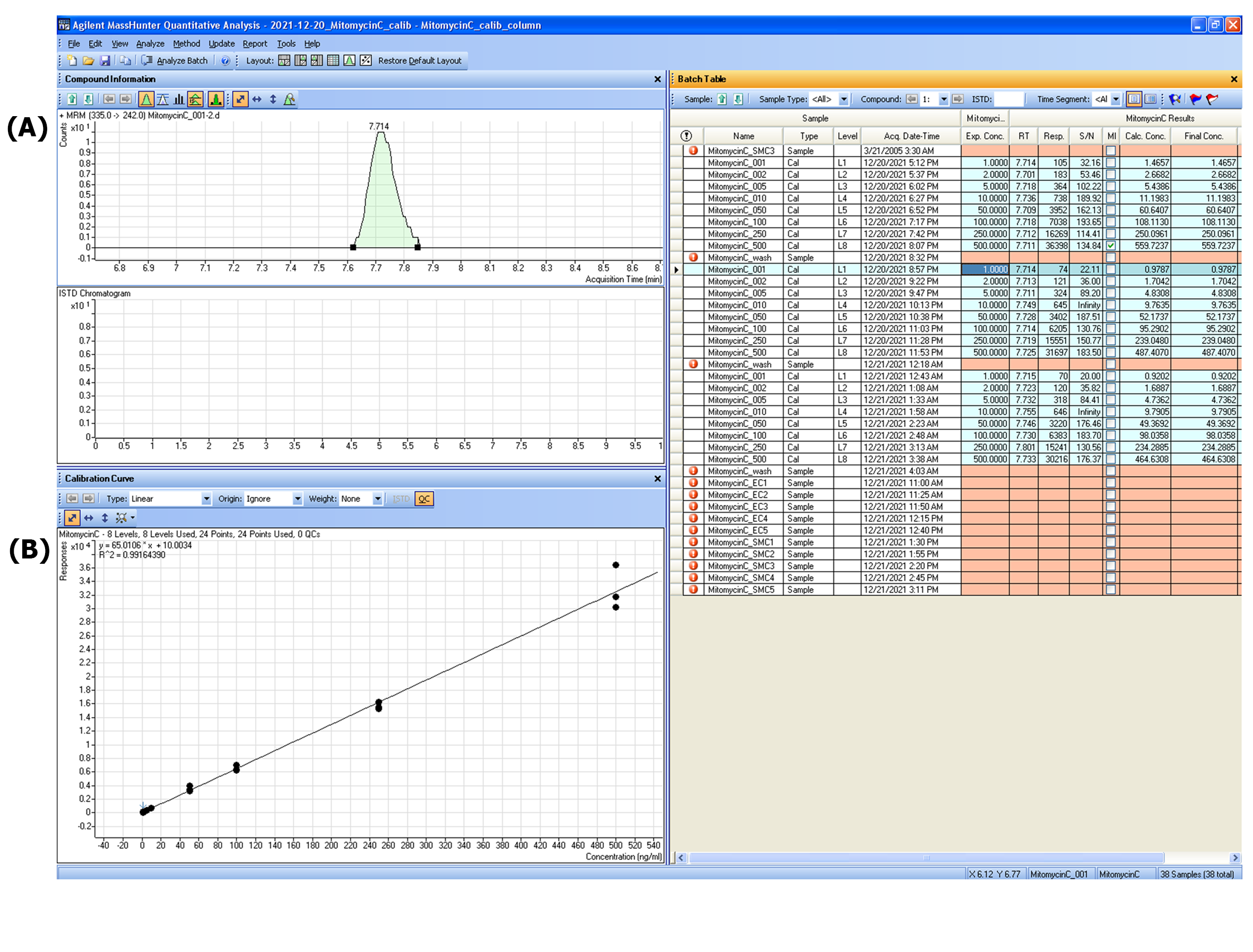

Supplement: Supplementary file 5 [file Image2.TIF]

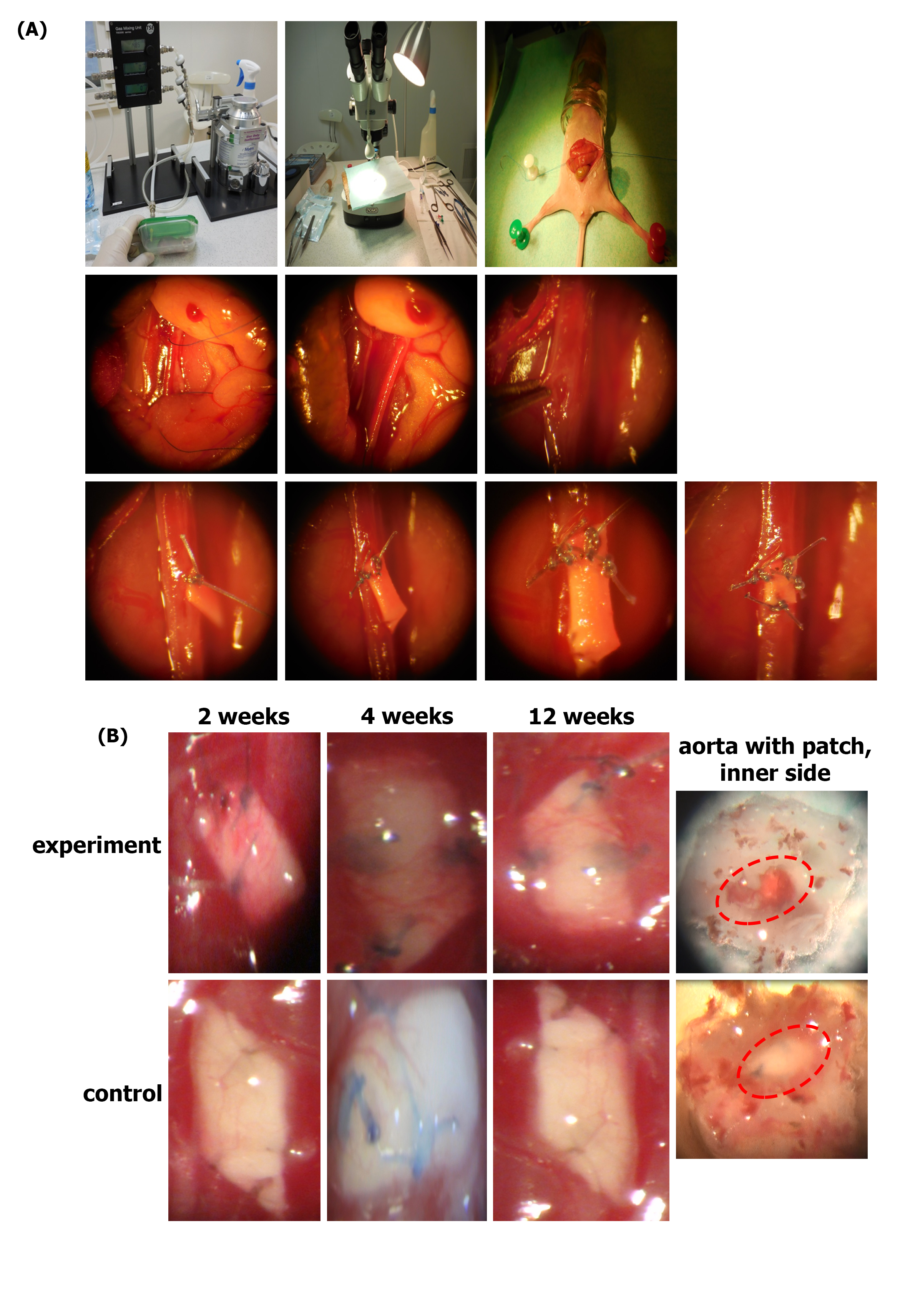

Supplement: Supplementary file 6 [file Image1.TIF]

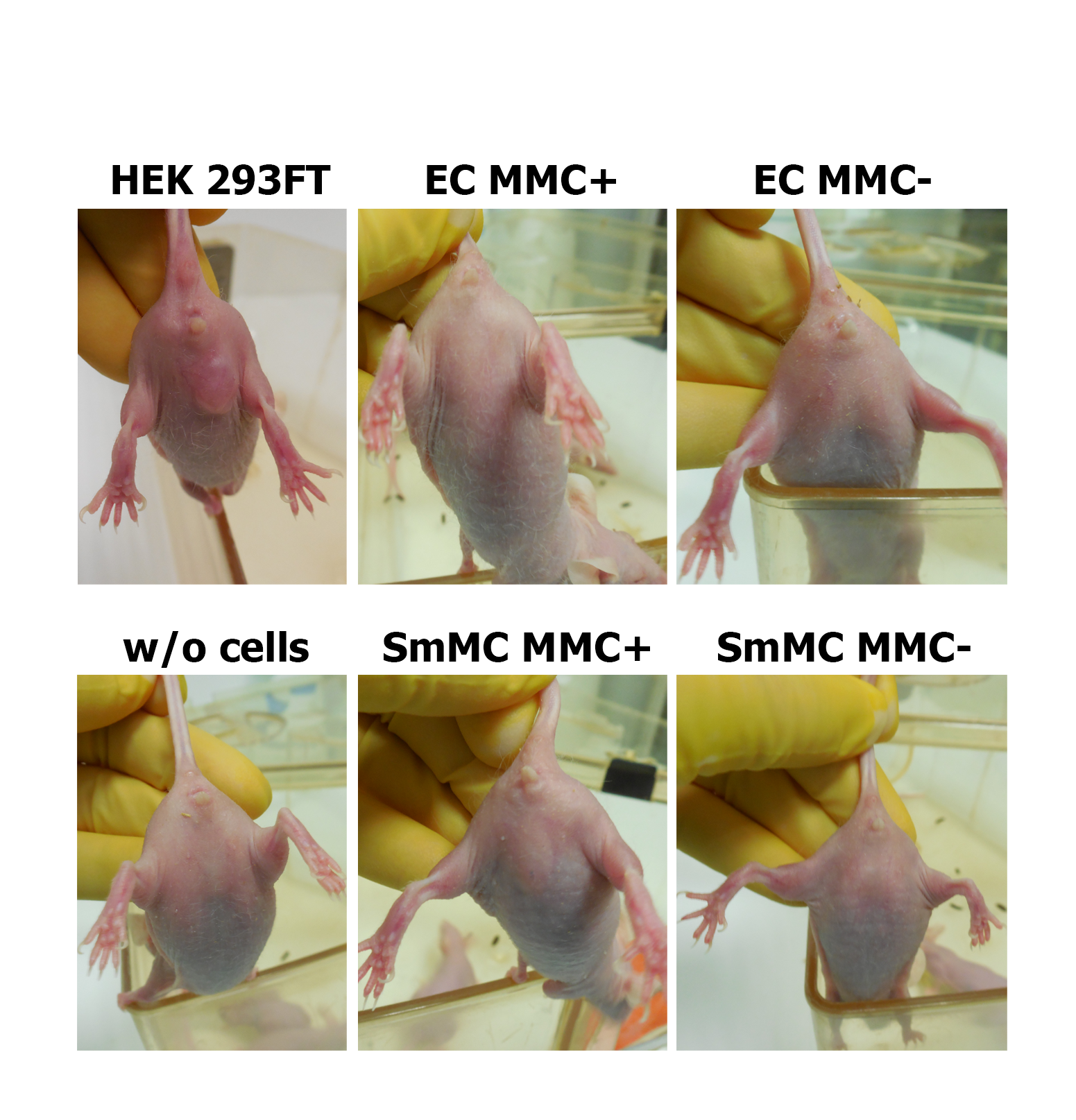

Supplement: Supplementary file 7 [file Image7.TIF]

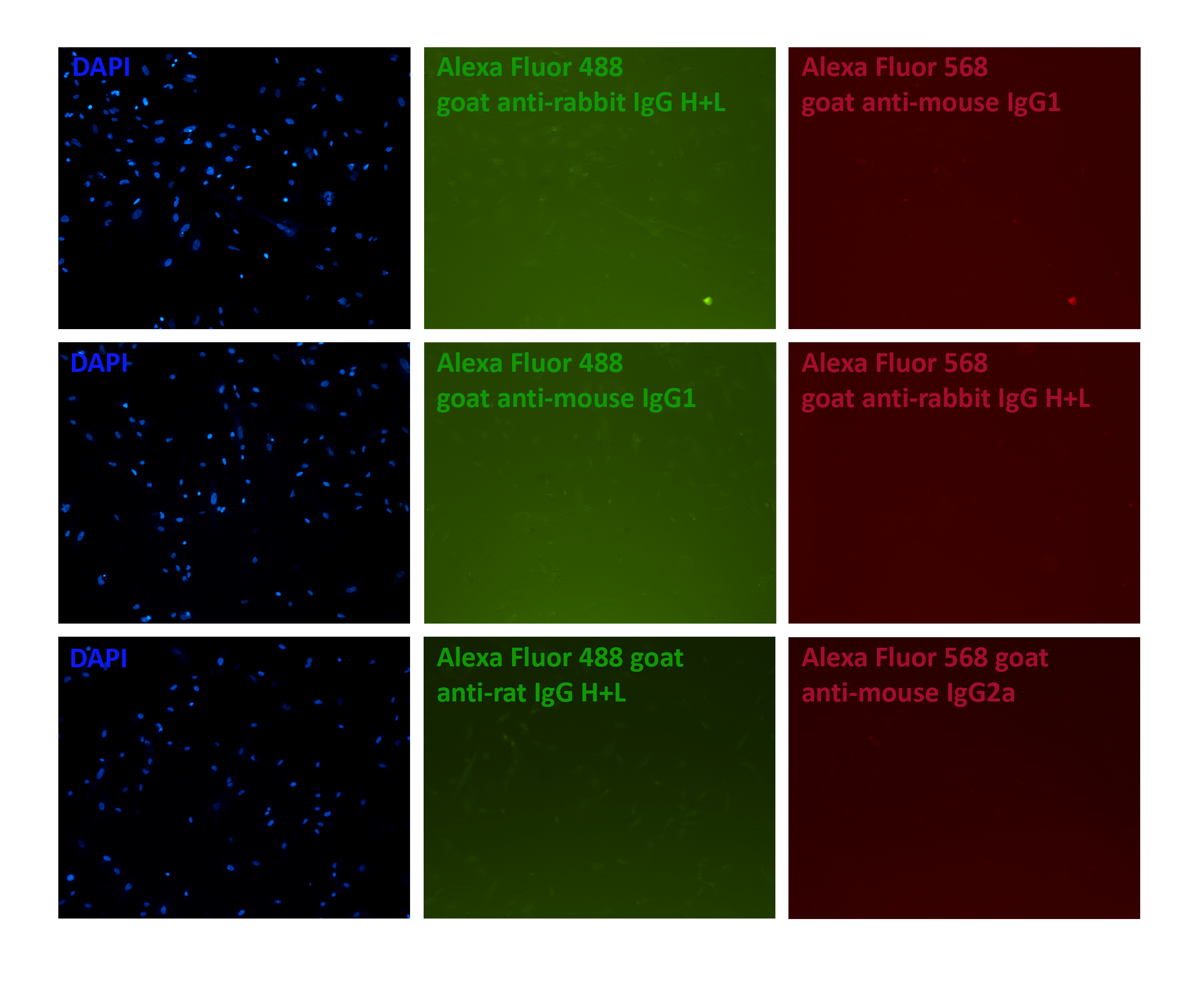

Supplement: Supplementary file 9 [file Image8.TIF]

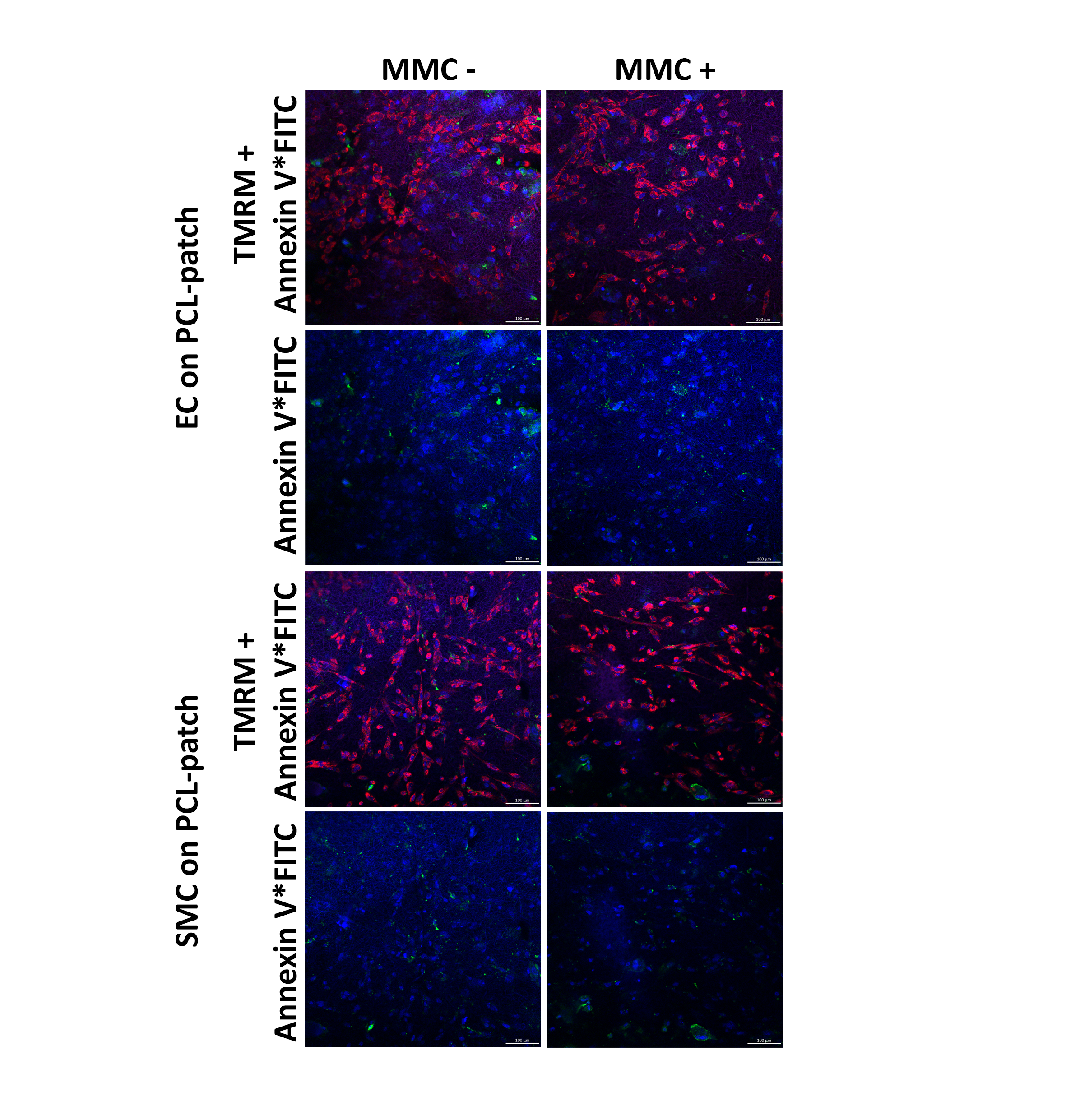

Supplement: Supplementary file 10 [file Image5.TIF]
