# Supplementary material for: Mitomycin-Treated Endothelial and Smooth Muscle Cells Suitable for Safe Tissue Engineering Approaches
Source: Front Bioeng Biotechnol. 2022 Mar 11;10:772981. doi: 10.3389/fbioe.2022.772981 (PMC8963790; doi:10.3389/fbioe.2022.772981)
Supplement: Supplementary file 11 [file DataSheet1.docx]

**Supplementary Table S1** CNVs detected in MMC-treated and untreated cells using molecular karyotyping

**Supplementary Video** Patch implantation.mov. The video shows the details of the patch implantation surgery and some of the next observations

**Supplementary Figure S1** Stages of aortic vascular patch implantation in SCID mice (A) and external neovascularization of the patch at control points (B). “Experiment” and “control” are groups of mice implanted with cell-seeded and unseeded patches, respectively. Aorta with patch, inner side explanted from a mouse after 4 weeks. The patch area contacted with blood is dotted red oval

**Supplementary Figure S2** The calibration curve for Mitomycin C, calculated from averaging three replicates of calibration solutions presented in the Batch Table. Window A shows the MRM signal from m/z 335 to 242 transition in the calibration solution with the lowest concentration 1 ng/mL of Mitomycin C.

**Supplementary Figure S3** The calibration curve for Mitomycin C, calculated from averaging three replicates of calibration solutions presented in the Batch Table. Window A shows the absence of MRM signal from m/z 335 to 242 transition in the SMC2 sample. The Batch table also contains no evidence of the transition of m/z from 335 to 242 in any of the SMC samples.

**Supplementary Figure S4** The calibration curve for Mitomycin C, calculated from averaging three replicates of calibration solutions presented in the Batch Table. Window A shows the absence of MRM signal from m/z 335 to 242 transition in the EC2 sample. The Batch table also contains no evidence of the transition of m/z from 335 to 242 in any of the EC samples.

**Supplementary Figure S5** The viability of cells seeded on PCL-patch assessed by TMRM and Annexin V*FITC staining. Nuclei are stained with NucBlue.

**Supplementary Figure S6** Karyotype analysis. Routine G-banding of MMC-untreated endothelial (A) and smooth muscle cells (B). C Absolute number of CNVs detected with molecular karyotyping in MMC-treated and untreated cells. D Percent of CNVs taking into account the statistical weight.

**Supplementary Figure S7** The area of subcutaneous injection of Matrigel with cells into the abdominal cavity. The tumor forms 1.5 months after the HEK293FT injection. 3 months after the injection of other cells, the tumor was not found

**Supplementary Figure S8** Representative images demonstrating negative control for autofluorescence with no primary antibody. 5-time over exposition, compared to detection with the primary antibody
